# Potential Mechanisms of Action of Chinese Patent Medicines for COVID-19: A Review

**DOI:** 10.3389/fphar.2021.668407

**Published:** 2021-07-15

**Authors:** Zhi-Hua Yang, Bin Wang, Qian Ma, Lin Wang, Ya-Xin Lin, Hai-Feng Yan, Zi-Xuan Fan, Hao-Jia Chen, Zhao Ge, Feng Zhu, Hui-Jie Wang, Bao-Nan Zhang, Hai-Dong Sun, Li-Min Feng

**Affiliations:** ^1^ First Teaching Hospital of Tianjin University of Traditional Chinese Medicine, Tianjin, China; ^2^ Tianjin University of Traditional Chinese Medicine, Tianjin, China; ^3^ Department of Traditional Chinese Medicine, Hebei North University, Zhangjiakou, China; ^4^ Department of Cardiology, Traditional Chinese Medicine Hospital of Tianjin Beichen District, Tianjin, China; ^5^ Tianjin Fourth Central Hospital, Tianjin, China; ^6^ Shenzhen Hospital Futian of Guangzhou University of Chinese Medicine, Shenzhen, China; ^7^ Second Affiliated Hospital of Tianjin University of Traditional Chinese Medicine, Tianjin, China

**Keywords:** COVID-19, SARS-CoV-2, traditional Chinese medicine, Chinese patent medicine, review

## Abstract

Coronavirus disease 2019 (COVID-19) is an emergent infectious pneumonia caused by severe acute respiratory syndrome coronavirus 2 (SARS-CoV-2), which is highly contagious and pathogenic. COVID-19 has rapidly swept across the world since it was first discovered in December 2019 and has drawn significant attention worldwide. During the early stages of the outbreak in China, traditional Chinese medicines (TCMs) were involved in the whole treatment process. As an indispensable part of TCM, Chinese patent medicines (CPMs) played an irreplaceable role in the prevention and treatment of this epidemic. Their use has achieved remarkable therapeutic efficacy during the period of medical observation and clinical treatment of mild, moderate, severe, and critical cases and during convalescence. In order to better propagate and make full use of the benefits of TCM in the treatment of COVID-19, this review will summarize the potential target of SARS-CoV-2 as well as the theoretical basis and clinical efficacy of recommended 22 CPMs by the National Health Commission and the Administration of TCM and local provinces or cities in the treatment of COVID-19. Additionally, the study will further analyze the drug composition, potential active ingredients, potential targets, regulated signaling pathways, and possible mechanisms for COVID-19 through anti-inflammatory and immunoregulation, antiviral, improve lung injury, antipyretic and organ protection to provide meaningful information about the clinical application of CPMs.

## Introduction

**FIGURE 1:**
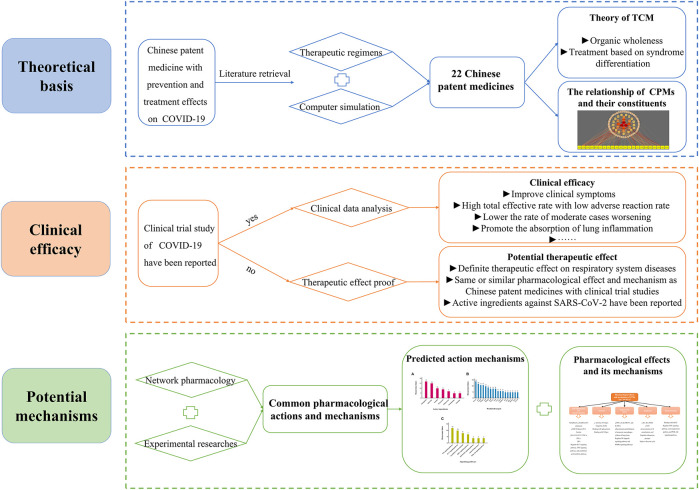
Graphical Abstract.

In December 2019, the first cases of unexplained viral pneumonia were reported in Wuhan, Hubei Province, China. According to the results of etiologic examinations, the pathogen that caused the disease was a novel coronavirus (Chan et al., 2020). On February 11, 2020, the novel coronavirus disease was officially named coronavirus disease 2019 (COVID-19) by the WHO (World Health Organization, 2020). On the same day, the International Committee on Taxonomy of Viruses (ICTV) gave the novel coronavirus its current name severe acute respiratory syndrome coronavirus 2 (SAR-CoV-2) (Coronaviridae Study Group of the International Committee on Taxonomy of Viruses, 2020). COVID-19 is an acute infectious disease caused by SARS-CoV-2 and is subsequent to severe acute respiratory syndrome (SARS) and Middle East respiratory syndrome (MERS) (Paules et al., 2020). It is highly infectious and is disseminating quickly worldwide. Lack of sufficient knowledge led to the rapid spread of the epidemic in the early stages. On January 30, 2020, the WHO declared the COVID-19 epidemic as a Public Health Emergency of International Concern (PHEIC) (World Health Organisation, 2020). In the subsequent months, the ongoing outbreak of COVID-19 progressed quickly and globally. The WHO defined the severity of the COVID-19 outbreak to be a global “pandemic” on March 12, 2020 (World Health Organisation, 2020). So far, COVID-19 has been mainly clinically treated by symptomatic therapies, supportive therapies, and symptom improvement, and at present, a few vaccines have been developed (Centers for Disease Control and Prevention, 2020). As one of the earliest countries to contain and tackle COVID-19, China has taken prompt and effective measures including isolation, elimination, personal protection, and treatment approaches using traditional Chinese medicine (TCM), Western medicine therapy, and a combination of the latter. In particular, in the absence of specific drugs and vaccines (Guo et al., 2020b), TCM has played an important role in the prevention and treatment of COVID-19 at various stages due to its characteristics of syndrome differentiation and treatment, integrated participation, and prevention before the disease escalates. In China, 92.58% of cases were treated by TCM, and the number of cases in which TCM participated in the clinical therapy and discharge has exceeded 70% with the proportion continuously increasing (State Administration of Traditional Chinese Medicine, 2020). The outbreak of COVID-19 has been well controlled in China for now, and TCM has contributed greatly (Xu and Zhang, 2020b). In particular, Chinese patent medicines (CPMs) have played an irreplaceable role and have provided unique advantages in the management of COVID-19. However, the underlying mechanisms of CPMs are still unclear. This review will summarize and analyze the possible active mechanisms of CPMs in the treatment of COVID-19. The findings reported may provide meaningful information for further study to investigate the mechanisms of CPMs as a therapeutic approach to overcoming COVID-19. Figure 1 shows the framework of this review.

## Potential Target for the Prevention and Inhibition of Severe Acute Respiratory Syndrome Coronavirus 2

SARS-CoV-2 is composed of four structural proteins and nonstructural proteins (NSP) (Gallagher and Buchmeier, 2001). Its structural proteins play an important role in the assembly of viruses and infection of hosts. Among them, the Spike (S) trimer is highly glycosylated to form the spikes on the surface of the virus, which is responsible for binding to the host cell receptor and allows the coronavirus to invade the host cell (Snijder et al., 2003). Studies have shown that angiotensin-converting enzyme 2 (ACE2) is a cell receptor which SARS-CoV-2 S glycoprotein can bind to (Zhou et al., 2020a). Alveolar type 2 (AT2) cells are the primary host cell of viral invasion due to it expressing higher levels of ACE2 protein. AT2 cells express higher levels of ACE2 protein. Viral replication induces an immune response in the body and produces excessive cytokines resulting in the formation of a cytokine storm after SARS-CoV-2 has entered the alveolar epithelial cells. This would cause inflammation and in turn lead to lung injury, acute respiratory distress syndrome (ARDS), and multiple organ failure and even death (Huang et al., 2020). Therefore, ACE2 is also a target molecule for lung damage caused by the virus (Kuba et al., 2005). Furthermore, cells in the heart, kidney, and other organs may become targets of viral invasion because of their high expression of ACE2. This will lead to damage to the lung, heart, kidney, and other important organs (Leng et al., 2020). Thus, disrupting the interaction between the SARS-CoV-2 S glycoprotein and this enzyme would prevent viral invasion. Nonstructural proteins of coronavirus participate in the transcription and replication of viral genomes, most of which are 3-chymotrypsin-like protease (3CLpro), Papain-like protease (PLpro), helicase, and 3–5′ exonuclease (Smith et al., 2013). The responsibility of SARS-CoV-2 3CLpro is to cleave polyproteins and generate functional enzymes. Without it, the entire replication architecture will not work properly (Loschwitz et al., 2021). So 3CLpro is considered to be a necessary target in the process of viral replication. Collectively, ACE2 and SARS-CoV-2 3CLpro are important targets for the prevention and inhibition of the virus, whose activity involves the invasion of host cells and intracellular replication of the virus (Song et al., 2018; Li et al., 2020a; Chen Y. W. et al., 2020; Wrapp et al., 2020).

## Theoretical Basis and Clinical Efficacy of Traditional Chinese Medicine in the Prevention and Treatment of Coronavirus Disease 2019

In the theory of TCM, COVID-19 belongs to the category of “plague,” given its feature of strong infectiousness and the resulting rapid epidemic. Exogenous pathogenic epidemic is the main etiology, and the virus target location is the lung. In addition, the basic pathogenesis is characterized by “dampness, heat, toxin, and stasis” (Chen et al., 2020a). Since ancient times, experts of TCM have accumulated rich clinical experience in the treatment of plagues. The advantage of TCM is that even if the pathogen is not clear, a set of corresponding prescriptions can be proposed based on clinical syndromes under the guidance of the theory of syndrome differentiation and treatment, which can alleviate the disease, shorten its course, and attenuate worsening or complications (Yang, 2020b). In this epidemic, TCM has exerted beneficial effects in a comprehensive way during the treatment of COVID-19. The concept of organic wholeness and the characteristics of treatment based on syndrome differentiation have contributed greatly to the therapy of COVID-19, especially in improving patients’ clinical symptoms, which is of great significance in enhancing survival rates (Li et al., 2020c).

**FIGURE 2:**
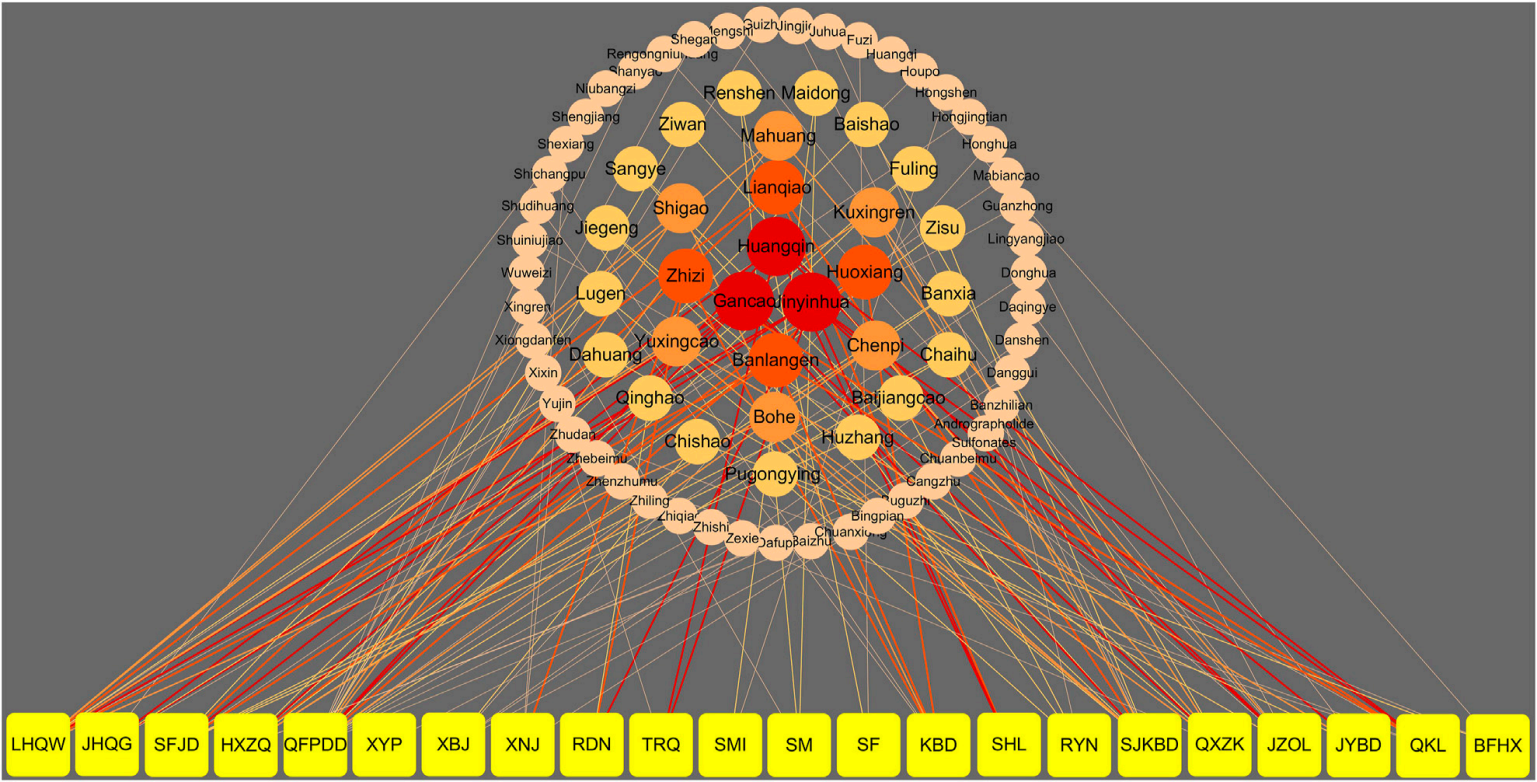
The relationship of the recommended CPMs and their constituents. Note: the yellow nodes represent CPMs. The other color nodes represent constituents of CPMs. The size and darkness of the nodes represent the appearance frequency of each constituent. The darkness of the nodes from dark to light and the size from large to small are illustrated in descending order of appearance frequency. The connecting lines indicate that each node is related. The width of the connecting lines was based on the appearance frequency of constituents, and the color and rule were same as the nodes. LHQW: Lianhua Qingwen Capsule; JHQG: Jinhua Qinggan Granules; SFJD: Shufeng Jiedu Capsule; HXZQ: Huoxiang Zhengqi Capsule; QFPDD: Qingfei Paidu decoction; XYP: Xiyanping Injection; XBJ:Xuebijing Injection; XNJ: Xingnaojing Injection; RDN: Reduning Injection; TRQ: Tanreqing Injection; SMI: Shenmai Injection; SM: Shengmai Injection; SF: Shenfu Injection; KBD: Kangbingdu Granules; SHL: Shuanghuanglian Oral Liquid; RYN: Reyanning Oral Liquid; SJKBD: Siji Kangbingdu Mixture; QXZK: Qingxuan Zhike Granules; JZOL: Jinzhen Oral Liquid; JYBD: Jinye Baidu Granules; QKL: Qingkailing Injection; BFHX: Bufei Huoxue Capsule.

At present, “Diagnosis and Treatment Protocol for COVID-19 (trial version 8)” formulated by the National Health Commission and the Administration of TCM and Chinese medicine prevention recommend 13 Chinese patent medicines for different stages (National Health Commission of the People’s Republic of China and National Administration of Traditional Chinese Medicine, 2020). During the medical observation period, the clinical manifestations were fatigue with gastrointestinal discomfort, and Huoxiang Zhengqi Capsule (pill, liquid, and oral liquid) was recommended. For the clinical manifestation of fatigue with fever, the recommended Chinese patent medicines are Jinhua Qinggan Granule, Lianhua Qingwen Capsule (granule), and Shufeng Jiedu Capsule (granule). During the clinical treatment period, Qingfei Paidu decoction can be used in the treatment of mild, moderate, and severe cases and can be used reasonably with the consideration of the actual conditions of critically ill patients. In addition, the corresponding prescriptions were recommended for different syndromes of mild, moderate, severe, and critical patients; Xiyanping Injection, Xuebijing Injection, Reduning Injection, Shengmai Injection, Shenfu Injection, Shenmai Injection, Tanreqing Injection, and Xingnaojing Injection are recommended for severe and critical patients. Besides, seven provincial and municipal diagnosis and treatment protocols involving nine types of CPMs were retrieved. The above 22 types of CPMs have been recommended as treatment schemes for the potential treatment of COVID-19 by using network pharmacology and molecular docking technology. The information of 22 CPMs is provided in Table 1. The details involving main findings, study design, severity classification, course of treatment, and clinical data for recommended CPMs are summarized in Table 2. Further, the relationship of CPMs and their constituents is depicted in Figure 2.

**TABLE 1:** Therapeutic regimens of recommended CPMs for COVID-19 in China.

No	Name of Chinese patent medicines	Clinical stage	Therapeutic regimen of COVID-19	Website
1	Lianhua Qingwen Capsule	Medical observation period	National Health Commission of the People’s Republic of China. Guideline on Diagnosis and Treatment of COVID-19 (trial 8th edition)	http://www.nhc.gov.cn/cms-search/xxgk/getManuscriptXxgk.htm?id=0a7bdf12bd4b46e5bd28ca7f9a7f5e5a
2	Jinhua Qinggan Granules	Medical observation period	National Health Commission of the People’s Republic of China. Guideline on Diagnosis and Treatment of COVID-19 (trial 8th edition)	http://www.nhc.gov.cn/cms-search/xxgk/getManuscriptXxgk.htm?id=0a7bdf12bd4b46e5bd28ca7f9a7f5e5a
3	Shufeng Jiedu Capsule	Medical observation period	National Health Commission of the People’s Republic of China. Guideline on Diagnosis and Treatment of COVID-19 (trial 8th edition)	http://www.nhc.gov.cn/cms-search/xxgk/getManuscriptXxgk.htm?id=0a7bdf12bd4b46e5bd28ca7f9a7f5e5a
4	Huoxiang Zhengqi Oral Capsule	Medical observation period	National Health Commission of the People’s Republic of China. Guideline on Diagnosis and Treatment of COVID-19 (trial 8th edition)	http://www.nhc.gov.cn/cms-search/xxgk/getManuscriptXxgk.htm?id=0a7bdf12bd4b46e5bd28ca7f9a7f5e5a
5	Qingfei Paidu decoction	Clinical treatment period—mild, moderate, severe, and critical cases	National Health Commission of the People’s Republic of China. Guideline on Diagnosis and Treatment of COVID-19 (trial 8th edition)	http://www.nhc.gov.cn/cms-search/xxgk/getManuscriptXxgk.htm?id=0a7bdf12bd4b46e5bd28ca7f9a7f5e5a
6	Xiyanping Injection	Clinical treatment period—severe case	National Health Commission of the People’s Republic of China. Guideline on Diagnosis and Treatment of COVID-19 (trial 8th edition)	http://www.nhc.gov.cn/cms-search/xxgk/getManuscriptXxgk.htm?id=0a7bdf12bd4b46e5bd28ca7f9a7f5e5a
7	Xuebijing Injection	Clinical treatment period—severe and critical cases	National Health Commission of the People’s Republic of China. Guideline on Diagnosis and Treatment of COVID-19 (trial 8th edition)	http://www.nhc.gov.cn/cms-search/xxgk/getManuscriptXxgk.htm?id=0a7bdf12bd4b46e5bd28ca7f9a7f5e5a
8	Xingnaojing Injection	Clinical treatment period—severe and critical cases	National Health Commission of the People’s Republic of China. Guideline on Diagnosis and Treatment of COVID-19 (trial 8th edition)	http://www.nhc.gov.cn/cms-search/xxgk/getManuscriptXxgk.htm?id=0a7bdf12bd4b46e5bd28ca7f9a7f5e5a
9	Reduning Injection	Clinical treatment period—severe and critical cases	National Health Commission of the People’s Republic of China. Guideline on Diagnosis and Treatment of COVID-19 (trial 8th edition)	http://www.nhc.gov.cn/cms-search/xxgk/getManuscriptXxgk.htm?id=0a7bdf12bd4b46e5bd28ca7f9a7f5e5a
10	Tanreqing Injection	Clinical treatment period—severe and critical cases	National Health Commission of the People’s Republic of China. Guideline on Diagnosis and Treatment of COVID-19 (trial 8th edition)	http://www.nhc.gov.cn/cms-search/xxgk/getManuscriptXxgk.htm?id=0a7bdf12bd4b46e5bd28ca7f9a7f5e5a
11	Shenmai Injection	Clinical treatment period—critical case	National Health Commission of the People’s Republic of China. Guideline on Diagnosis and Treatment of COVID-19 (trial 8th edition)	http://www.nhc.gov.cn/cms-search/xxgk/getManuscriptXxgk.htm?id=0a7bdf12bd4b46e5bd28ca7f9a7f5e5a
12	Shengmai Injection	Clinical treatment period—critical case	National Health Commission of the People’s Republic of China. Guideline on Diagnosis and Treatment of COVID-19 (trial 8th edition)	http://www.nhc.gov.cn/cms-search/xxgk/getManuscriptXxgk.htm?id=0a7bdf12bd4b46e5bd28ca7f9a7f5e5a
13	Shenfu Injection	Clinical treatment period—critical case	National Health Commission of the People’s Republic of China. Guideline on Diagnosis and Treatment of COVID-19 (trial 8th edition)	http://www.nhc.gov.cn/cms-search/xxgk/getManuscriptXxgk.htm?id=0a7bdf12bd4b46e5bd28ca7f9a7f5e5a
14	Kangbingdu Granules	Medical observation period; clinical treatment period—mild case	Guangdong Provincial COVID-19 TCM Therapeutic Regime (trial version II)	http://szyyj.gd.gov.cn/zwgk/gsgg/content/post_2902010.html
15	Shuanghuanglian Oral Liquid	Medical observation period; clinical treatment period—mild and moderate cases	Beijing Municipal COVID-19 TCM Preventive and Therapeutic Regime (trial version V)	http://zyj.beijing.gov.cn/sy/tzgg/202006/t20200616_1926196.html
16	Reyanning Mixture	Clinical treatment period—mild case	Shanxi provincial COVID-19 TCM therapeutic regime (trial version II)	http://atcm.shaanxi.gov.cn/sy/tzgg/202002/t20200202_2048353.html
17	Siji Kangbingdu Mixture	Clinical treatment period—mild case	Shanxi Provincial COVID-19 TCM Therapeutic Regime (trial version II)	http://atcm.shaanxi.gov.cn/sy/tzgg/202002/t20200202_2048353.html
18	Qingxuan Zhike Granules	Clinical treatment period—mild case	Hunan Provincial COVID-19 newborn infants Preventive and Therapeutic Regime (trial version I)	http://www.hnysfww.com/article.php?id = 1820
19	Jinzhen Oral Liquid	Clinical treatment period—mild and moderate cases	The 24th press conference on COVID-19 prevention and control held by the Information Office of Guangdong Provincial Government	http://www.gd.gov.cn/gdywdt/zwzt/yqfk/fbh/content/post_2924024.html
20	Jinye Baidu Granules	Clinical treatment period—mild and moderate cases	TCM Diagnosis and Treatment Scheme and Preventive Scheme for Novel Coronavirus Infected Pneumonia of Tongji Hospital, Tongji Medical College, Huazhong University of Science & Technology	https://www.tjh.com.cn/html/2020/0208/28991.shtml
21	Qingkailing Injection	Clinical treatment period—severe case	TCM Diagnosis and Treatment Scheme and Preventive Scheme for Novel Coronavirus Infected Pneumonia of Tongji Hospital, Tongji Medical College, Huazhong University Of Science & Technology	https://www.tjh.com.cn/html/2020/0208/28991.shtml
22	Bufei Huoxue Capsule	Convalescence	Guizhou Provincial COVID-19 TCM Preventive and Therapeutic Reference Regime (version II)	http://atcm.guizhou.gov.cn/xwzx/tzgg/202002/t20200219_50073831.html

**TABLE 2:** Summary of clinical efficacy of CPMs for COVID-19.

No	Name of Chinese patent medicines	Main findings	Study design	Severity classification	Course of treatment	Clinical data	References
1	Lianhua Qingwen Capsule (LHQW)	① Improve clinical symptoms of fever, fatigue, cough, expectoration, shortness of breath, moist crackles, chest distress, and appetite loss. ② Enhance the effective rate of cardinal symptom. ③ Reduce the proportion of normal to heavy.	Retrospective study; LHQW group: LHQW + conventional western therapy (*n* = 63) vs. control group: conventional western therapy (*n* = 38)	Medical observation period	10 days	① LHQW 86.7% vs. control 67.7%, *p* < 0.05	Lu et al. (2020)
						② LHQW 55.6% vs. control 30.6%, *p* < 0.05	
						③ LHQW 82.5% vs. control 58.6%, *p* < 0.05	
						⑤ Shortness of breath: LHQW 68.2% vs. control 20.0%, *p* < 0.05; moist crackles: LHQW 56.0% vs. control 20.0%, *p* < 0.05	
						⑩ Fever: LHQW 6 days vs. control 7 days, *p* = 0.17	
						⑭ LHQW 6.40% vs. control 15.8%, *p* > 0.05	
			Retrospective study; LHQW group: LHQW + conventional western therapy (*n* = 51) vs. control group: conventional western therapy (*n* = 51)	Ordinary	7 days	① LHQW 83.7% vs. control 61.0%, *p* < 0.05	Cheng et al. (2020)
						② LHQW 61.3% vs. control 34.3%, *p* < 0.05	
						③ LHQW 62.2% vs. control 35.9%, *p* < 0.05	
						⑤ Expectoration: LHQW 55.0% vs. control 15.8%; shortness of breath: LHQW 61.5% vs. control 14.3%, *p* < 0.05; chest distress: LHQW 54.6% vs. control 15.8%, *p* < 0.05; appetite loss: LHQW 34.8% vs. control 7.70%, *p* < 0.05	
						⑥ LHQW 54.9% vs. control 45.1%, *p* > 0.05	
						⑫ LHQW 86.3% vs. control 68.6%, *p* < 0.05	
						⑭ LHQW 7.80% vs. control 21.6%, *p* < 0.05	
2	Jinhua Qinggan Granules (JHQG)	① Significantly reduce the clinical symptoms of fever, cough, fatigue, and expectoration. ② Relieve the anxiety of patients.	Randomized controlled trial; JHQG group: JHQG + conventional western therapy (*n* = 82) vs. control group: conventional western therapy (*n* = 41)	Mild	5 days	① JHQG 80.3% vs. control 53.1%, *p* = 0.0050	Duan et al. (2020)
						② JHQG 66.1% vs. control 42.9%, *p* = 0.038	
						③ JHQG 77.6% vs. control 53.8%, *p* = 0.028	
						④ JHQG 85.3% vs. control 46.2%, *p* = 0.017	
						⑪ Compared with the control group, JHQG group significantly decrease, *p* < 0.01	
3	Shufeng Jiedu Capsule (SFJD)	① Significantly improve the clinical symptoms, such as cough, expectoration, fatigue, chest distress, and shortness of breath. ③ Promote the absorption of lung inflammation. ④ Significantly shorten the symptoms improvement time and negative conversion time of the clinical.	Retrospective study; SFJD group: SFJD + arbidol hydrochloride capsule (*n* = 40) vs. control group: arbidol hydrochloride capsule (*n* = 30)	Mild and ordinary	10 days	⑧ SFJD 11.9 ± 3.21 vs. control 9.32 ± 3.03, *p* < 0.05	Qu et al. (2020)
						⑩ Compared with the control group, dry cough, nasal congestion, runny nose, pharyngeal pain, fatigue and diarrhea significantly improved (*p* < 0.05) in the SFJD group	
			Retrospective study; SFJD group: SFJD + conventional western therapy (*n* = 34) vs. control group: conventional western therapy (*n* = 34)	Ordinary	7 days	② SFJD 91.3% vs. control 54.2%, *p* < 0.05	Chen et al. (2020c)
						③ SFJD 100% vs. control 70.6%, *p* < 0.05>	
						④ SFJD 100% vs. control 37.5%, *p* < 0.05	
						⑤ Chest distress: SFJD 100% vs. control 57.1%, *p* < 0.05; shortness of breath: SFJD 90.9% vs. control 45.5%, *p* < 0.05	
						⑥ SFJD 91.2% vs. control 70.6%, *p* < 0.05	
						⑦ The level of lymphocytes significantly increased (*p* < 0.05) and the level of C-reactive protein significantly decreased (*p* < 0.05) in both two groups; while procalcitonin and D-dimer significantly decreased (*p* < 0.05) in the SFJD group	
4	Huoxiang Zhengqi Oral Capsule (HXZQ)	① Significantly improve clinical symptoms such as fever, cough, fatigue, and white greasy coating. ② Lower the rate of moderate cases worsening. ③ Improve the clinical cure rate.	Case series; COVID-19 patients treated by HXZQ and conventional western therapy (*n* = 11)	Ordinary	9–33 days	① 100%	Yang et al. (2020a)
						② 60.0%	
						③ 100%	
						⑤ 64.0%	
						⑫ 100%	
						⑭ 9.09%	
5	Qingfei Paidu decoction (QFPD)	① Significantly improve clinical symptoms and TCM syndrome. ③ Improve the negative psychology and decrease risks of complications.	Randomized controlled trial QFPD group: QFPD + conventional western therapy (*n* = 70) vs. control group: conventional western therapy (*n* = 70)	Ordinary	10 days	⑦ QFPD 95.7% vs. control 85.7%, *p* < 0.05	Wang et al. (2021)
						⑨ Compared with the control group, QFPD group significantly shorter, *p* < 0.05	
						⑫ QFPD 98.6% vs. control 90.0%, *p* < 0.05	
						⑬QFPD 1.43% vs. Control 12.9%, *p* < 0.05	
			Retrospective study; QFPD group: QFPD + conventional western therapy (*n* = 104) vs. control group: conventional western therapy (*n* = 125)	Ordinary	30 days	⑥ QFPD 89.4% vs. control 71.2%, *p* < 0.001	Zeng et al. (2020)
						⑧ QFPD 5.36 ± 1.25 vs. control 10.6 ± 2.62, *p* < 0.001	
						⑨ QFPD 24.6 ± 2.31 vs. control 29.4 ± 2.47, *p* < 0.001	
						⑪ Compared with the control group, QFPD group significantly decrease, *p* < 0.05	
						⑮ QFPD 51.0% vs. Control 70.4%, *p* = 0.0040	
6	Xuebijing Injection (XBJ)	① Promote the absorption of pulmonary infection.	Retrospective study; XBJ group: XBJ + conventional western therapy (*n* = 22) vs. control group: conventional western therapy (*n* = 22)	Ordinary	7 days	⑥ XBJ 95.5% vs. control 68.2%, *p* = 0.017	Zhang et al. (2020a)
						⑫ XBJ 68.2% vs. control 50.0%, *p* = 0.0010	
			Randomized controlled trial; XBJ group: XBJ + conventional western therapy (*n* = 10) vs. control group: conventional western therapy (*n* = 10)	Mild	3 days	⑦ The level of lymphocyte count, CRP, and ESR in XBJ group were better than those of the control group, *p* > 0.05	Liu et al. (2020a)
7	Reyanning Mixture (RYN)	① Improve the clinical symptoms of COVID-19 patients, such as sore throat, cough, fatigue, headache, and chest tightness. ② Promote the improvement of chest CT. ③ Shorten the duration of fever and improve the novel coronavirus nucleic acid conversion rate.	Randomized controlled trial; RYN group: RYN + conventional western therapy (*n* = 26) vs. control group: conventional western therapy (*n* = 23)	Ordinary	7 days	①-⑤ Except cough and fatigue, other symptoms disappeared in the RYN group, and the disappearance rate of symptoms such as dry throat, cough, fatigue, chest tightness, and headache was statistically significant compared with the control group (*p* < 0.05)	Yang et al. (2020c)
						⑥ RYN 88.5% vs. control 74.0%, *p* > 0.05	
						⑧ RYN 96.2% vs. control 60.9%, *p* < 0.01	
						⑩ Fever: RYN 5 days vs. control 3 days, *p* > 0.05	
8	Jinye Baidu Granules (JYBD)	Lower the rate of moderate and mild cases worsening	Retrospective study; JYBD group: JYBD + conventional western therapy (*n* = 27) vs. Control group: methylprednisolone + conventional western therapy (*n* = 38)	Mild and ordinary	5–7 days	⑭ JYBD 7.40% vs. control 42.1%, *p* < 0.05	Yu et al. (2020a)
9	Reduning Injection (RDN)	① Significantly reduce the levels of inflammatory factors	Retrospective study; RDN group: RDN + methylprednisolone (*n* = 21) vs. control group: methylprednisolone (*n* = 26)	Severe	5–7 days	⑦ Compared with control group after treatment, the levels of WBC, IL6, IL17, and CRP in RDN group were significantly lower (*p* < 0.05), and the level of IL4 in group A was significantly higher (*p* < 0.05)	Qin et al. (2020)
		② Reduce the length of hospital stay and ICU stay				⑨ The length of hospitalization in RDN group was significantly shorter (*p* < 0.05)	
10	Tanreqing Injection (TRQ)	Improve symptoms, block deterioration and promote rehabilitation	Retrospective study; TRQ group: TRQ+α-interferon + conventional western therapy (*n* = 58) vs. control group: conventional western therapy (*n* = 60)	All confirmed stages	7 days	⑩ The symptom score in the TRQ group was significantly higher (*p* < 0.05)	Huang et al. (2020b)
						⑫ TRQ 93.1% vs. control 80.0%, *p* < 0.05	

Note: Clinical data①–⑮: ① disappearance rate of fever; ② disappearance rate of cough; ③ disappearance rate of fatigue; ④ disappearance rate of expectoration; ⑤ disappearance rate of other signs and symptoms; ⑥ the improvement rate of pulmonary CT; ⑦ the recovery level of inflammatory indexes; ⑧ the negative conversion rate (time) of nucleic acid; ⑨ the length of hospitalization; ⑩ the duration of main symptoms; ⑪ the anxiety level; ⑫ total effective rate; ⑬ the adverse reaction rate; ⑭ aggravation rate; ⑮ complication rate.

Table 2 shows ten CPMs recommended for the treatment of COVID-19, including 14 completed studies in our review. Among 14 studies, three studies involved mild patients, seven studies involved ordinary patients, two studies involved both mild and ordinary patients, only one study involved severe patients, and one study did not report the stage of severity of COVID-19. There are 15 indexes used to evaluate the therapeutic effect of Chinese patent medicines. The total effective rate, aggravation rate, and adverse reaction rate are mainly used to evaluate the efficacy and safety of Chinese patent medicines. In addition, it also involves the disappearance rate of main symptoms, the duration of main symptoms, improvement rate of pulmonary CT, recovery rate of inflammation level, negative conversion rate (time) of nucleic acid, and other secondary indexes. The result shows the total effective rates of LHQW (Cheng et al., 2020), HXZQ (Yang et al., 2020a), QFPD (Wang et al., 2021), and TRQ (Huang et al., 2020b) were above 85% and the treatment effect is obvious. Although the total effective rate of Xuebijing was only 68.2%, there was a significant difference compared with the control group. The adverse reaction rate and the aggravation rate were low, less than 10% (Cheng et al., 2020; Yang H. B. et al., 2020; Yu H. Y. et al., 2020; Lu et al., 2020; Wang et al., 2021), indicating that those CPMs can improve the efficacy with a good safety profile. These clinical trials found that these drugs can significantly improve the clinical symptoms caused by COVID-19. For patients of mild and moderate cases, it is necessarily important to improve the main symptoms such as fever, cough, fatigue, and expectoration, which avoid the transformation from mild to severe. CPMs contribute the most to the improvement of fever symptoms, the disappearance rate of fever is more than 80% (Duan et al., 2020), and the duration of fever was shorter in the treatment group (Cheng et al., 2020; Lu et al., 2020; Yang, 2020b). However, CPMs in relieving cough symptoms are weak, and the disappearance rate of cough is only 55% (Lu et al., 2020). By observing the improvement rate of pulmonary CT and the recovery level of inflammatory indexes, we can further know the therapeutic effect of CPMs on COVID-19. SFJD (Chen et al., 2020c), QFPD (Zeng et al., 2020), and XBJ (Zhang et al., 2020a) can significantly improve the absorption of lung CT lesions, LHQW (Cheng et al., 2020) and RYN (Yang et al., 2020c) also have this therapeutic effect, but there is no significant difference. Before and after the treatment, the difference of the oxygenation indexes and inflammatory factors such as white blood cell (WBC), lymphocytes (LYMPH), erythrocyte sedimentation rate (ESR), interleukin (IL), tumor necrosis factor-α (TNF-α), C-reactive protein (CRP), and procalcitonin (PCT) was compared between the two groups, which in the treatment group was better than that in the control group (Liu K. F. et al., 2020; Qin et al., 2020; Chen et al., 2020c; Wang et al., 2021). Besides, SFJD (Qu et al., 2020), QFPD (Zeng et al., 2020), and RYN (Yang et al., 2020c) can shorten the negative conversion rate (time) of nucleic acid.

At present, the randomized controlled trials of some drugs have not been completed (such as Shengmai Injection and Bufei Huoxue Capsule) or are still in progress (such as Xiyanping Injection and Shuanghuanglian Oral Liquid), so the effectiveness of drugs in the treatment of COVID-19 cannot be determined temporarily (Wang et al., 2020a). However, because of the need for treatment, the above drugs have been widely used in the treatment of patients with COVID-19 in accordance with the strategy of “New uses of old drugs” and “Treating different diseases with the same method” in the TCM theory. The active component of the Xiyanping Injection is the total sulfonate of andrographolide, and it has the function of clearing heat and detoxification and antibacterial and anti-inflammatory and definite therapeutic effect on respiratory system diseases (Cai et al., 2020). Shuanghuanglian Oral Liquid (Zheng et al., 2020b) and Xingnaojing Injection (Xie et al., 2020) also have the function of regulating inflammatory factors and relieving inflammation reaction. This is one of the therapeutic mechanisms of COVID-19, which was treated by avoiding the cytokine storm. Shenfu Injection, Shengmai Injection, and Shenmai Injection can enhance the immune function and hypoxia tolerance of myocardial cells, but the anti-inflammatory effect of Shengmai Injection is stronger than that of the other two (Zhang and Yang, 2008; Li et al., 2016). Early experiments *in vitro* antibiosis and *in vivo* animal studies showed (Wu et al., 2018) that Kangbingdu Granules had an antibacterial effect *in vitro* and antiviral, antipyretic, and anti-inflammatory effects *in vivo*. Liu et al. (2020b) conducted a meta-analysis for Siji Kangbingdu Mixture efficacy and safety in the treatment of children respiratory system infections. The result shows that Siji Kangbingdu Mixture can shorten the time of fever, cough, and sore throat, the efficacy effect and safety of Siji Kangbingdu Mixture were significantly better than those of ribavirin or interferon. Qingxuan Zhike Granules in the treatment of children with upper respiratory infection (Zhang et al., 2015) and cold of wind-heat (Du et al., 2021) can effectively improve the clinical symptoms, especially for cough, sputum, and pharyngeal symptoms. Jinzhen Oral Liquid (Yu, 2019) has antipyretic, anti-inflammatory, antitussive, and antiviral pharmacological effects, and it is mainly used in the treatment of children with various respiratory diseases in clinical. Qingkailing Injection can promote the release of antipyretic substances and regulate body temperature by inhibiting endogenous pyrogen and endotoxic fever (Zhao et al., 2019). Bufei Huoxue Capsules (Zhou et al., 2020b) can improve lung ventilation function, reduce blood viscosity, and increase blood oxygen content to play a role in the treatment of chronic respiratory diseases. These drugs can improve clinical efficiency and reduce the incidence of adverse reactions in respiratory diseases (State Administration of Traditional Chinese Medicine, 2020; Xu and Zhang, 2020b). But, due to differences in usage, dosage, and application population, the adverse reactions may have new changes. So clinical trials should be completed as soon as possible (Hu and Zhang, 2020).

Figure 2 shows that *Lonicera japonica* Thunb, *Glycyrrhiza uralensis* Fisch, *Scutellaria baicalensis* Georgi, *Gardenia jasminoides* Ellis, Isatidis Radix, Forsythia suspensa Vahl, and Agastache rugosa are frequently used among all the recommended CPMs (chose only if frequency ≥4 for CPMs). These drugs are widely used in the treatment of COVID-19 for its function of clearing away heat and toxin (Chen et al., 2020b; Yu Y. R. et al., 2020). Zhou et al. (2021) suggested that *Lonicera japonica* Thunb extract significantly increased body weight, organ index, serum cytokines (interleukin, tumor necrosis factor, and interferon-γ), secretory immunoglobulin A (sIgA), and the activity of natural killer (NK) cells and cytotoxic lymphocytes (CTL) of colitis ulcerative mouse. Besides, it can also reduce the apoptosis of splenic lymphocytes in colitis ulcerative mouse (Nomura et al., 2019). It has been found Glycyrrhizic acid can inhibit the replication of the virus and interfere with the cycle of virus adsorption and penetration. Yu et al. (2020b) indicated that Glycyrrhizic acid could inhibit the binding of RBD part of S protein and ACE2 by the MTT assay to play antiviral effect. Some researchers used *Scutellaria baicalensis* Georgi for antibacterial test *in vitro*. Some experiment results showed (Li et al., 2013) that *Scutellaria baicalensis* Georgi has a strong inhibitory effect on *Staphylococcus aureus*, *Escherichia coli*, *Pseudomonas aeruginosa*, and other viruses. Moreover, baicalein, an active ingredient of *Scutellaria baicalensis* Georgi, has a strong inhibitory effect on HIV. *Gardenia jasminoides* Ellis is an important medicine to cure epidemic diseases. It can significantly inhibit the lung inflammation of mice induced by influenza virus, significantly reduce the mortality of mice infected by influenza virus, prolong the survival time, and reduce the level of NO in serum. It also can inhibit the cytopathy caused by H1N1, PIV-1, RSV, HSV-1, and HSV-2 *in vitro* (Wang et al., 2006). Isatidis Radix, with an antiviral pharmacological effect, can inhibit a variety of influenza viruses, such as H1N1, H3N2, and H9N2, and also has a good anti-inflammatory effect. It can inhibit virus-induced inflammatory response by regulating the NF-kB signaling pathway and the TLR3 signaling pathway (Huang et al., 2019). The antibacterial and antiviral effects of *Forsythia suspensa* were evaluated by Shi et al. (2013) in combination with the existing *in vitro* and *in vivo* studies. The study found that *Forsythia suspensa* has a good inhibitory effect on *Escherichia coli*, *Staphylococcus aureus*, *Salmonella typhi*, *Escherichia coli*, endotoxin, *Staphylococcus* epidermidis, influenza A virus, human cytomegalovirus, Japanese encephalitis virus, respiratory syncytial virus, and simple cytomegalovirus. The extract of *Agastache rugosa* has the ability of immunoregulation. The results showed (Chen et al., 2020d) that the volatile oil of *Agastache rugosa* leaves had different effects on immune cells at different times. The serum containing *Agastache rugosa* leaves could significantly activate the white blood cells, macrophages, and lymphocytes of mice and promote the immune function of the body.

## Mechanisms of Traditional Chinese Medicine in the Therapy of Coronavirus Disease 2019

According to the discussion of clinical trials, the above 22 kinds of CPMs have good or potential clinical effects for the prevention and treatment of patients with COVID-19. Therefore, it is particularly important to explore the mechanism of action of CPMs for COVID-19 by computer simulation and *in vivo* or *vitro* experimental studies. We reviewed the potential mechanisms of action of CPMs and tried to find more research clues for CPMs with definite curative effect and provide theoretical support for CPMs without clinical research report. The results involving constituents, predicted active ingredients, predicted targets, and regulated signaling pathways for recommended CPMs are summarized in Table 3. The detailed mechanisms are as follows.

**TABLE 3:** Summary of potential action mechanisms of CPMs for COVID-19.

No	Name of Chinese patent medicine	Latin name of composition	Chinese name of composition	Predicted active ingredient	Predicted target	Regulated signaling pathways	References
1	Lianhua Qingwen Capsule	Ephedra sinica Stapf, Rheum palmatum L., Lonicera japonica Thunb, Mentha canadensis L., Glycyrrhiza glabra L., Rhodiola crenulata (Hook.f. & Thomson) H.Ohba, Cyrtomium Fortunei J.Sm., Gypsum Fibrosum, Pogostemon cablin (Blanco) Benth, Prunus armeniaca L., Houttuynia cordata Thunb, Forsythia suspensa (Thunb.) Vahl, Isatis tinctoria L.	Mahuang, Dahuang, Jinyinhua, Bohe, Gancao, Hongjingtian, Guanzhong, Shigao, Huoxiang, Kuxingren, Yuxingcao, Lianqiao, Banlangen	Kaempferol, quercetin, luteolin, glycyrrhetinic acid, indigo, β-sitosterol, etc.	PTGS2, IL6, CASP3, MAPK1, EGFR, ACE2, TNF, IL1B, MAPK8, CCL2, IL10, IL2, IFNG, etc.	AGE-RAGE signaling pathway, IL17 signaling pathway, JAK-STAT signaling pathway, TNF signaling pathway	Ling et al. (2020); Wang et al., (2020c); Wang et al. (2020g)
2	Jinhua Qinggan Granules	Lonicera japonica Thunb, Artemisia annua L., Arctium lappa L., Scutellaria baicalensis Georgi, Fritillaria thunbergii Miq.	Qinghao, Jinyinhua, Niubangzi, Huangqin, Zhebeimu	Quercetin, kaempferol, naringenin, isorhamnetin, formononetin, 7-methoxy-2-methyl, β-sitosterol, isoflavone, licochalcone B, glyasperin C, licochalcone a, 3′-methoxyglabridin, anhydroicaritin, stigmasterol, etc.	AKT1, RELA, PTGS2, HSP90AB1, HSP90AA1, PTGS1, NCOA2, CALM1, AR, NOS2, ESR1, etc.	TNF signaling pathway, IL17 signaling pathway, PI3K-Akt signaling pathway, HIF-1 signaling pathway	Shen et al. (2020); Jimilihan et al. (2020)
3	Shufeng Jiedu Capsule	Glycyrrhiza glabra L., Forsythia suspensa (Thunb.) Vahl, Isatis tinctoria L., Reynoutria japonica Houtt, Patrinia scabiosifolia Link, Phragmites australis (Cav.) Trin. ex Steud., Verbena officinalis L., Bupleurum chinense DC.	Huzhang, Baijiangcao, Lianqiao, Lugen, Mabiancao, Chaihu, Banlangen, Gancao	Quercetin, 6-(3-oxoindolin-2-ylidene) indolo (2,1-b), β-sitosterol, kaempferol, luteolin, quinazolin-12-one, bicuculline, isorhamnetin, physciondiglucoside, dihydroverticillatine, licoisoflavanone, 5,7,4’-trihydroxy-8-methoxyflavone, acacetin	PTGS2, ESR1, AR, NOS2, PTGS1, NCOA2, F10, ALB, PPARG, PRSS1, SCN5A, IL6, IL1B, FOS, CCL2, MAPK8, MAPK1, MAPK14, CASP3, etc.	Human cytomegalovirus infection, Kaposi sarcoma-associated herpesvirus infection, IL17 signaling pathway, small-cell lung cancer	Cao et al. (2020); Xu et al. (2020c)
4	Huoxiang Zhengqi Oral Liquid	Glycyrrhiza glabra L., Magnolia officinalis Rehder & E.H.Wilson, Perilla frutescens (L.) Britton, Areca catechu L., Smilax glabra Roxb, Atractylodes Lancea (Thunb.) DC., Paeonia lactiflora Pall, Citrus × aurantium L., Pinellia ternata (Thunb.) Makino, Pogostemon cablin (Blanco) Benth.	Houpo, Banxia, Zisu, Dafupi, Fuling, Cangzhu, Baishao, Chenpi, Gancao, Huoxiang	Quercetin, isorhamnetin, irisolidone, kaempferol, wogonin, baicalein	PTGS2, HSP90AB1, AR, CAMSAP2, PPARG, NOS2, etc	TNF signaling pathway, HIF-1 signaling pathway, toxoplasmosis, bladder cancer, prostate cancer, pancreatic cancer, pathways in cancer	Deng et al. (2020)
5	Qingfei Paidu decoction	Ephedra sinica Stapf, Glycyrrhiza glabra L., Gypsum Fibrosum, Scutellaria baicalensis Georgi, Bupleurum chinense DC., Smilax glabra Roxb, Citrus × aurantium L., Pogostemon cablin (Blanco) Benth, Neolitsea cassia (L.) Kosterm, Alisma plantago-aquatica subsp. orientale Sam, Polyporus umbellatus (Pers.) Fr., Atractylodes macrocephala Koidz, Pinellia ternata (Thunb.) Makino, *Zingiber officinale* Roscoe, Aster tataricus L.f., Tussilago farfara L., Polygonum aviculare L., Asarum sieboldii Miq., Dioscorea oppositifolia L., Prunus armeniaca L.	Mahuang, Gancao, Xingren, Shigao, Guizhi, Zexie, zhuling, Baizhu, Fuling, Chaihu, Huangqin, Banxia, Shengjiang, Ziwan, Donghua, Shegan, Xixin, Shanyao, Zhishi, Chenpi, Huoxiang	Baicalin, Glycyrrhizic acid, hesperidin, hyperoside	AKT1, TNF-α, IL6, PTGS2, HMOX1, IL10, TP53	NOD-like receptor signaling, Toll-like receptor signaling, cytokine–cytokine receptor interaction, chemokine signaling, Th17 cell differentiation, IL17 signaling pathway, NF-kappa B signaling pathway, TNF signaling pathway, necroptosis, apoptosis, HIF-1 signaling pathway, mitophagy	Zhao et al. (2021)
6	Xiyanping Injection	Andrographolide sulfonates.	Andrographolide sulfonates	Andrographolide	COX-2, IL6, IL1β, TNF, MAPK1, MAPK4, MAPK8, MAPK14, etc.	—	Cai et al. (2020)
7	Xuebijing Injection	Conioselinum anthriscoides, Carthamus tinctorius L., Angelica sinensis (Oliv.) Diels, Paeonia veitchii Lynch, Salvia miltiorrhiza Bunge.	Chishao, Danggui, Honghua, Danshen, Chuanxiong	Quercetin, gallic acid, luteolin, rosmarinic acid, rutin, kaempferol, chlorogenic acid, tanshinone II A, hydroxysafflor yellow A, paeoniflorin	PTGS2, PTGS1, CASP3, RELA, TNF, MAPK1, IL2, IL6, IL10, etc.	IL17 signaling pathway, hepatitis C, hepatitis B, toxoplasmosis	Kong et al. (2020a)
8	Xingnaojing Injection	Curcuma aromatica Salisb., Moschus berezovskii Flerov, Dryobalanops aromatica C.F.Gaertn., Gardenia jasminoides J.Ellis.	Shexiang, Yujin, Bingpian, Zhizi	Chlorogenin, kaempferol	PARP1, PTGS2, MMP9, CDK2, ADORA2A, ALOX5, GSK3B, etc.	Hepatitis B, pathways in cancer, TNF signaling pathway, HIF-1 signaling pathway, VEGF signaling pathway, apoptosis	Xie et al. (2020)
				5-Hydroxy-6,7,3′,4′,5′-pentamethoxyflavone, isokaempferol, morin, gardenin, quercetin, artemisetin, genistein, dryobalanone, curcumin, elemicin, etc.			
9	Reduning Injection	Lonicera japonica Thunb, Artemisia annua L., Gardenia jasminoides J.Ellis.	Zhizi, Qinghao, Jinyinhua	Apigenin, quercetin, kaempferol, luteolin, stigmasterol, β-sitosterol, isorhamnetin, chrysoeriol	RELA, MAPK1, MAPK14, MAPK8, IL6, CASP8, CASP3, IL1B, STAT1, TNF, CCL2, etc.	AGE-RAGE signaling pathway, human cytomegalovirus infection, TNF signaling pathway, IL17 signaling pathway	Pu et al. (2020); Gao et al. (2020); Chen et al. (2020e)
10	Tanreqing Injection	Lonicera japonica Thunb, Scutellaria baicalensis Georgi, Forsythia suspensa (Thunb.) Vahl, Saiga tatarica Linnaeus, Fel Ursi.	Huangqin, Xiongdanfen, Jinyinhua, Liaoqiao, Lingyangjiao	Quercetin, baicalein, luteolin, wogonin, kaempferol, scutellarin, baicalin, oroxylin-7-O-glucuronide, forsythin, forsythiaside E, ursodeoxycholic acid, chenodeoxycholic acid, etc.	TNF, EGFR, NOS3, PTGS2, IL2, GABBR1, MAPK14, ADRB2, REN, VCAM1, ACHE, PTPRC, IL6, IL1B, MAPK1, IL10, IL4, CXCL8, MAPK14, MAPK8, STAT1, CASPS3, TP53, IFNG, RB1, CXCL2, etc.	IL17 signaling pathway, T-cell receptor signaling pathway, arachidonic acid metabolic pathway, cAMP signaling pathway, PI3K-Akt signaling pathway, influenza A	Kong et al. (2020b)
11	Shenmai Injection	Ophiopogon japonicus (Thunb.) Ker Gawl., Talinum paniculatum (Jacq.) Gaertn.	Hongshen, Maidong	Ophiopogonin D’, ophiopogonin D, ginsenoside rg 2, methyl ophiopogonanone A, ginsenoside Rb 2, ginsenoside R0, ophiopogon A, sanchinoside rd, ophiopogonanone E, ginsenoside re, etc	IL6, GAPDH, ALB, TNF, MAPK1, MAPK3, TP53, EGFR, CASP3, CXCL8	HIF-1 signaling pathway, TNF signaling pathway, sphingolipid signaling pathway, toll-like signaling pathway, neurotrophin signaling pathway	Han et al. (2020)
12	Shengmai Injection	Ophiopogon japonicus (Thunb.) Ker Gawl., Schisandra chinensis (Turcz.) Baill., Talinum paniculatum (Jacq.) Gaertn.	Maidong, Hongshen, Wuweizi	Schisanlactone E, stigmasterol, N-trans-feruloyltyramine, β-sitosterol, angeloylgomisin O, gomisin-A, gomisin R, changnanic acid, kadsulactone, kadsulignan B, etc.	CASP3, CASP8, PTGS2, BCL2, BAX, PRKCA, PTGS1, PIK3CG, F10, NOS3, DPP4	AGE-RAGE signaling pathway, p53 signaling pathway, small-cell lung cancer, apoptosis	Wang et al. (2020d)
					NOS2, TLR9, ACE, ICAM1, PRKCE, etc.		
13	Shenfu Injection	Cyperus rotundus L., Panax ginseng C.A.Mey.	Fuzi, Renshen	kaempferol, ginsenoside rh2, β-sitosterol, bisindigotin, stigmasterol, etc.	CASP3, MAPK8, IL1B, PPARG, PTGS2, CASP8, HMOX1, ICAM1, IFNG, NOS2, RELA, STAT1, MAPK14, PPARG, NOS3, etc.	AGE-RAGE signaling pathway, IL17 signaling pathway, C-type letcin receptor, HIF-1 signaling pathway	Li et al. (2020e); Wang et al. (2020d)
14	Kangbingdu Granules	Gypsum Fibrosum, Isatis tinctoria L., Pogostemon cablin (Blanco) Benth, Rehmannia glutinosa (Gaertn.) DC., Acorus gramineus Aiton, Gardenia jasminoides J.Ellis.	Zhizi, Shichangpu, Banlangen, Shudihuang, Huoxiang, Shigao	Angiotensin-converting enzyme 2, bicuculline, luteolin, quercetin, kaempferol, β-sitosterol, sitosterol, stigmasterol, stigmasterol, irisolidone, 8-isopentenyl-kaempferol, etc.	PTGS2, HSP90AB1, PTGS1, TP53, NCOA2, AKT1, JUN, TNF, ESR1, SCN5A, etc.	Prostate cancer, small-cell lung cancer, TNF signaling pathway, pathways in cancer	Yao et al. (2020)
15	Shuanghuanglian Oral Liquid	Lonicera japonica Thunb, Forsythia suspensa (Thunb.) Vahl, Scutellaria baicalensis Georgi.	Lianqiao, Huangqin, Jinyinhua	Quercetin, β-sitosterol, luteolin, stigmasterol, kaempferol, neobaicalein, dihydrooroxylin A	CASP3, TP53, MAPK8, IL6, MAPK1, MAPK1, CCL2, etc.	TNF signaling pathway, HIF-1 signaling pathway, pathway in cancer, tuberculosis, hepatitis C, pertussis, salmonella infection, influenza A, herpes simplex virus infection	Zheng et al. (2020b)
16	Reyanning Oral Liquid	Reynoutria japonica Houtt, Patrinia scabiosifolia Link, Taraxacum mongolicum Hand.-Mazz., Scutellaria barbata D. Don.	Pugongying, Banzhilian, Huzhang, Baijiangcao	Apigenin, chrysin-5-methylether, catechin, 7,2′-dihydroxy-5,8-dime thoxyflavone, 7-hydroxy-5,8-dimethoxy-2-phenyl-chromone, 5,7-dihydroxy-8-methoxy-2-(2-methoxyphenyl)chromone, etc.	CCL2, CD40LG, CXCL10, CXCL8, IFNG, IL10, IL13, IL1B, IL2, IL6, etc.	IL17 signaling pathway, cytokine–cytokine receptor interaction pathway	Wang et al. (2020e)
17	Siji Kangbingdu Mixture	Mentha canadensis L., Glycyrrhiza glabra L., Prunus armeniaca L., Houttuynia cordata Thunb, Forsythia suspensa (Thunb.) Vahl, Phragmites australis (Cav.) Trin. ex Steud., Perilla frutescens (L.) Britton, Morus alba L., Chrysanthemum × morifolium (Ramat.) Hemsl., Platycodon grandiflorus (Jacq.) A.DC., Nepeta cataria L.	Sangye, Zisu, Juhua, Lugen, Jingjie, Yuxingcao, Kuxingren, Gancao, Bohe, Lianqiao, Jiegeng	Quercetin, kaempferol, luteolin, rutin, naringenin	PTGS1, ADRB2, JUN, IL6, IL1B, IL10, IFNG, ACHE, IL2, etc.	IL17 signaling pathway, cytokine–cytokine receptor interaction, viral protein interaction with cytokine and cytokine receptor, arachidonic acid metabolism	Liu et al. (2020b)
18	Qingxuan Zhike Granules	Mentha canadensis L., Glycyrrhiza glabra L., Prunus armeniaca L., Paeonia lactiflora Pall, Citrus × aurantium L., Aster tataricus L.f., Morus alba L., Platycodon grandiflorus (Jacq.) A.DC.	Kuxingren, Chenpi, Baishao, Ziwan, Sangye, Gancao, Bohe, Jiegeng, Zhiqiao	Quercetin, kaempferol, luteolin, 7-methoxy-2-methyl, formononetin, etc.	PTGS2, ESR1, HSP90AA1, CALM1, AR, etc.	Small cell lung cancer, non-small-cell lung cancer, T-cell receptor signaling pathway	Zong et al. (2020)
19	Jinzhen Oral Liquid	Ephedra sinica Stapf, Rheum palmatum L., Lonicera japonica Thunb, Scutellaria baicalensis Georgi, Saiga tatarica Linnaeus, Lapis Chiloriti, Bovis Calculus Artif Actus, Fritillaria cirrhosa D.Don.	Mengshi, Chuanbeimu, Lingyangjiao, Rengongniuhuang, Huangqin, Jinyinhua, Dahuang, Mahuang	Isoglabrolide, glabrolide, ebeiedinone, desoxo-glabrolid-acetate, peimisine, verticinone, imperialine, ussuriedinone, euchrenone A5, quercetin, kaempferol, naringenin, baicalein, etc.	mTOR, JAK3, ACE2, TNF-α, AKT2, PIK3CA, MEK1, BRD2, ACE, ANPEP, MAPK3, MAPK8, IL6, CASP3, IL10, MAPK1, CXCL8, CCL2, etc.	PI3K-Akt signaling pathway, Jak-STAT signaling pathway, TNF signaling pathway, MAPK signaling pathway	Tao et al. (2020)
20	Jinye Baidu Granules	Lonicera japonica Thunb, Houttuynia cordata Thunb, Taraxacum mongolicum Hand.-Mazz., Isatis tinctoria L.	Daqingye, Pugongying, Yuxingcao, Jinyinhua	Kaempferol, glycyrol, indirubin, etc.	TNF, IL1, IL6, IL8, PTGS2, PTGS1, NOS3, PPARG, etc.	—	Shi et al. (2020a)
21	Qingkailing Injection	Lonicera japonica Thunb, Isatis tinctoria L., Scutellaria baicalensis Georgi, Gardenia jasminoides J.Ellis, Cornu Margaritifera, Cornu Bubali.	Shuiniujiao, Zhenzhumu, Jinyinhua, Zhizi, Banlangen, Huangqin	Acacetin, syrigin, kaempferol, quercetin, emodin, luteolin, apigenin, etc.	CASP3, CASP8, FASLG, EGFR, CYCS, EGF, BCL2L1, IL4, PPARG, MCL1, etc.	TNF signaling pathway, Fc epsilon RI, PI3K-Akt signaling pathway	Zhang et al. (2020c)
22	Bufei Huoxue Capsule	Paeonia veitchii Lynch, Astragalus mongholicus Bunge, Cullen corylifolium (L.) Medik.	Huangqi, Buguzhi, Chishao	Quercetin, kaempferol, baicalein, 7-O-methylisomucronulatol, formononetin, β-sitosterol, stigmasterol, isorhamnetin, ellagic acid, 3,9-di-O-methylnissolin, etc.	IL6, MAPK8, PTGS2, PTGS1, NCOA2, NOS2, RELA, CXCL8, IL10, MAPK1, FOS, CASP3, MAPK14, IRF1, IL1B, CD14, IL1A, etc.	Pertussis, Chagas disease, TNF signaling pathway, Salmonella infection, tuberculosis	Guo et al. (2020a)

### Chinese Patent Medicine Has Been Reported in Clinical Trials

Lianhua Qingwen Capsule has good antiviral, anti-inflammatory, and immunomodulatory effects. Ling et al. (2020) reported that Lianhua Qingwen Capsule could act on SARS-CoV-2 *via* multiple components, targets, and pathways. Its main ingredients kaempferol, quercetin, and luteolin have better combinations with Mpro, while indigo, glycyrrhetinic acid, and stigmasterol can target the ACE2; thereby, it is possible to cure COVID-19. Li et al (2020b) found that Lianhua Qingwen Capsule could significantly inhibit the replication of SARS-CoV-2 in cells and reduce the expression of viral particles and the upregulation of inflammatory cytokines TNF-α, IL6, MCP-1, and IP-10 caused by SARS-CoV-2 infection, in a dose-dependent manner in host cells. It has a comparable antiviral potency against the SARS-CoV-2 with an IC50 value of 411.2 μg/ml. Ling et al. (2020), Li et al. (2020b), and Wang et al. (2020c) speculated that Lianhua Qingwen Capsule activity against COVID-19 was exerted through its active ingredients quercetin, luteolin, kaempferol, glycyrrhetinic acid, stigmasterol, and indigo, which influenced inflammatory cytokine targets to regulate the AGE-RAGE, IL17, JAK-STAT, and TNF signaling pathways and other signaling pathways to restrain the viability of SARS-CoV-2 and improve the clinical symptoms of patients with COVID-19. New studies have proved (Li et al., 2019) that Lianhua Qingwen Capsule can effectively reduce the motility chemotaxis potential of macrophages in the acute lung injury (ALI) model and reduce the recruitment of monocyte macrophages by downregulating MCP-1. *In vivo*, Lianhua Qingwen Capsule can effectively inhibit the infiltration of macrophages, further reduce the edema of lung tissue, reduce the temperature fluctuation caused by ALI, and alleviate the lung pathological injury of ALI.

By using network pharmacology and molecule docking analyses, Gong et al. (2020), Shen et al. (2020), and Jimilihan et al. (2020) identified formononetin, stigmasterol, 3-methoxy-glycerol, anhydroicaritin, beta-sitosterol, crude-glycerin, glycyrrhizin B, and other key compounds from Jinhua Qinggan Granules as having a certain degree of affinity with the SARS-CoV-2 3CL hydrolase and ACE2—which might regulate downstream TNF, IL17, PI3K/Akt, and HIF-1 signaling pathways and other signaling pathways and regulate their activity on targets such as PTGS2, HSP90AB1, HSP90AA1, PTGS1, NCOA2, AKTI, and RELA—for the prevention of COVID-19-related lung injury. Mao et al. (2020) and Lin et al. (2020) reported that the mechanism of action of Jinhua Qinggan Granules in the treatment of COVID-19 involved multiple targets and multiple pathways related to antiviral activity, immune regulation, inflammation inhibition, and apoptosis regulation.

Cao et al. (2020) reported that 6-(3-oxoindolin-2-ylidene)indolo(2,1-b)quinazolin-12-one, bicuculline, physciondiglucoside, dihydroverticillatine, and licoisoflavanone are compounds present in Shufeng Jiedu Capsule that could regulate the signaling pathways involved in human cytomegalovirus infection, Kaposi’s sarcoma-associated herpesvirus infection, the IL17 signaling pathway, and small cell lung cancer, which could be active to treat COVID-19 by binding with SARS-CoV-2 3CL hydrolase and ACE2. Xu et al. (2020c) speculated that Shufeng Jiedu Capsule might regulate key targets such as IL6, IL1β, MAPK8, MAPK1, MAPK14, CCL2, CASP3, FOS, and ALB mainly through flavonoids such as quercetin, luteolin, wogonin, acacetin, isorhamnetin, 5,7,4’-trihydroxy-8-methoxyflavone, kaempferol, and phytosterol, which in turn influence the inflammatory response, oxidative stress injury, apoptosis, pulmonary fibrosis, and other processes that reduce lung injury induced by COVID-19. Furthermore, Huang et al. (2020a) and Xu et al. (2020a) reported that Shufeng Jiedu Capsule could treat COVID-19 through multiple components, multiple targets, and multiple pathways of TCM, and its potential mechanism might involve immune-inflammatory response and antiviral activity. Its active ingredients have the potential to help people resist infection by SARS-CoV-2 as they interfere with the binding of SARS-CoV-2 to ACE2. Liu et al. (2016) suggested that Shufeng Jiedu Capsule has a significant antipyretic effect, which could reduce the levels of PGE2, TNF-α, IL1α, IL6, and IL1β, reduce the heat production, and increase the content of AVP.

Also using molecule docking analyses, Deng et al. (2020) found that the core compounds in Huoxiang Zhengqi Oral Liquid such as quercetin, isorhamnetin, irisolidone, kaempferol, wogonin, and baicalein were similar in affinity to currently recommended drugs for the treatment of COVID-19 such as remdesivir, ribavirin, and ritonavir. Of these, isorhamnetin, quercetin, and irisolidone had the strongest affinity. These compounds may interfere with ACE2 binding to PTGS2, HSP90AB1, AR, CAMSAP2, and other targets that regulate multiple signaling pathways and thus exert a preventive or therapeutic effect on COVID-19. Du et al. (2020) reported that elicorice glycoside E, naringenin, robinin, [(2R)-7-hydroxy-2-(4-hydroxyphenyl)chroman-4-one], and kaempferol strongly bind to 3CL protease and have been proposed as potential inhibitors of 3CL protease. Furthermore. the antiviral pathway of Huoxiang Zhengqi might be exerted through the PI3K-Akt signaling pathway and its downstream effects on viral replication.

Zhao et al. (2021) analyzed the treatment mechanism of Qingfei Paidu decoction from molecular, pathway, and network levels and conducted *in vitro* experiments to verify. The result showed that the main component of Qingfei Paidu decoction—baicalin—can suppress NF-κB signaling and downregulate the expression of IL6 and TNF-α and CCL2 in macrophage-like cell line RAQ264.7. In addition, Qingfei Paidu decoction can inhibit the activity of PTGS2 and PGE2 production in order to exhibit the effects of immune regulation and regulate ribosomal proteins related to the viral replication so that it inhibited the growth and production of virus.

In the clinical treatment of patients with COVID-19, Xuebijing Injection mainly inhibited IL6, TNF-α, MCP1, mip2, and IL10 to inhibit inflammatory response (Huang et al., 2020a). Xuebijing Injection could inhibit respiratory viruses while relieving inflammation, delay pathological changes in the lungs, and protect from liver damage caused by viruses or antiviral drugs. It can reduce the apoptosis of tubular cells during sepsis, improve the expression of apoptosis-related proteins Bcl-2 and Bax, and alleviate kidney injury (Sun et al., 2017). It could also inhibit bacterial infections, improve body immunity, and reduce cardiovascular damage (Kang, 2020; Shi X. et al., 2020). Zheng et al. (2020a) reported that hyperoside, salvianolic acid C, salvianolic acid A, cynaroside, salvianolic acid B, and paeoniflorin were the main components of Xuebijing Injection and had a good affinity with SARS-CoV-2 3CL hydrolase and its human receptor, ACE2. Two mechanisms were potentially involved in Xuebijing treatment of SARS-CoV-2-induced pneumonia. First, its main activity could be mainly exerted through the regulation of the human immune-inflammatory response to protect important organs, and second, Xuebijing Injection might also act on the essential proteins of the virus, 3CLpro and ACE2, to induce antiviral effects. Xuebijing Injection presents the characteristics of multiple components and activity against multiple targets and pathways in treating COVID-19, based on its overall synergy (Kong et al., 2020a; He et al., 2020). Chen et al. (2018) showed that 18 ml/kg Xuebijing Injection could stimulate Treg differentiation and moderately inhibit Th17 differentiation, effectively prevent neutrophil infiltration into lung and kidney, and improve its survival rate in this septic shock model.

Reyanning Mixture is recommended by the Diagnosis and Treatment Protocol for COVID-19 (trial version 2) statement issued by the Shanxi Province for the therapy of clinically mild and severe cases. Reyanning Mixture exerted beneficial effects in treating coronavirus pneumonia mice by its activity on the lungs. The result showed that Reyanning Mixture can reduce the lung index of coronavirus pneumonia mice with pestilence attacking the lung, significantly increase the percentage of CD8^+^ T and CD4^+^ T lymphocytes in peripheral blood of model mice, increase the percentage of total B lymphocytes, reduce virus load in lung tissue, reduce the levels of TNF-α, IFN-γ, IL6, and IL10 in the lung tissue, and reduce the content of motilin in the serum of model mice. It manifested obvious therapeutic effects by improving lung lesions, enhancing gastrointestinal function, improving autoimmune function, and reducing the expression of inflammatory factors *in vivo*, which provide a rationale for future clinical applications of the active compounds (Bao et al., 2020). Wang et al. (2020e) reported that the core compounds in Reyanning Mixture such as apigenin, chrysin-5-methylether, and catechin act on CD40LG, CXCL10, CXCL8, IL10, IL2, IL6, and other targets involved in the IL17 signaling pathway and cytokine–cytokine receptor interaction pathway.

Jinye Baidu Granules are suitable for the treatment of viral pneumonia, acute upper respiratory tract infection, influenza, and other viral and bacterial diseases. It has been used for the prevention and control of SARS virus infection during the SARS outbreak in 2003 and achieved good results. At present, it has been listed as a reserved drug for the prevention and control of COVID-19 in Hubei, Guangdong, Hebei, Fujian, and Anhui Provinces. In the presence of a cytokine storm causing DIC, sepsis, Jinye Baidu Granules particles exert a protective effect on liver microsomal activity, calcium homeostasis, in addition to resisting free radical production and activity, and reducing levels of circulating or tissue inflammatory cytokines (such as TNF-α, IL1, IL6, and IL8) and inhibiting dysregulated TNF-α release, which lead to the reduction of vascular endothelial cells and organ damage and prevention of the activation of the blood coagulation system (Shi et al., 2020a).

Pu et al. (2020), Gao et al. (2020), and Chen et al. (2020e) reported that Reduning Injection might act on targets such as inflammatory cytokines and MAPKs through its active ingredients quercetin, kaempferol, beta-sitosterol, luteolin, isorhamnetin, and chrysoeriol, to regulate the AGE-RAGE, human cytomegalovirus infection, TNF, and IL17 signaling pathways and to treat COVID-19 patients. In addition, the antipyretic mechanism of Reduning Injection is related to inflammatory mediators such as IL1, ET-1, IL6, and PGE 2 and regulating endogenous pyrogen (Wang et al., 2013).

Kong et al. (2020b) showed that kaempferol, quercetin, baicalein, luteolin, and wogonin, which are the active compounds of Tanreqing Injection, showed good affinity toward SARS-CoV-2 3CL hydrolase. The molecular mechanism of Tanreqing Injection in the treatment of COVID-19 involved the synergistic features of multiple components, targets, and pathways of TCM.

### Chinese Patent Medicines with Potential Therapeutic Effect

Cai et al. (2020) reported andrographolide, a main component of Xiyanping Injection, had potential antiviral effects in the treatment of COVID-19. It could reduce the level of inflammation in patients, improve respiratory symptoms, inhibit concurrent bacterial infection, and improve the immune response. The research showed (Yang and Luo, 2017) that andrographolide can reduce the level of Th17 and Th2 type cells and play an immunomodulatory role. At the same time, it would not induce similar immunosuppressive effects of steroids, and the incidence of adverse reactions was low. *Andrographis paniculata* Nees, which has the same active ingredient as Xiyanping Injection, can inhibit LPS-induced hyperthermia in rabbits by reducing the levels of 5-HT and increasing the levels of cAMP in the brain (Xu et al., 2019). In addition, andrographolide can reduce the inflammatory damage of the liver by downregulating NF-κB signaling pathway to reduce the expression of inflammatory factors, including TNF-α and IL6.

Xie et al. (2020) discovered the core compounds in Xingnaojing Injection including chlorogenin, 3-methylkempferol, kaempferol, morin, 5-hydroxy-6,7,3′,4′,5′-pentamethoxyflavone, gardenin, quercetin, dryobalanone, artemisetin, genistein, curcumin, and elemicin. These compounds might interfere with various signaling pathways by acting on the key targets, such as PARP1, PTGS2, MMP9, CDK2, ADORA2A, ALOX5, and GSK3B. They may also regulate the inflammatory response, apoptosis, oxidative stress, angiogenesis, and the other processes to alleviate the neurological damage sometimes caused by SARS-CoV-2. Further, they inhibited viral replication and prevented infection of the host cell by binding with Mpro, and the ACE2 complex. These activities implied that Xingnaojing Injection might have a positive therapeutic effect on the neurological damage caused by SARS-CoV-2.

Shenfu Injection, Shenmai Injection, and Shengmai Injection are recommended for severe patients, which have a good role in relieving cytokine storm. Their main components are similar, which can inhibit inflammatory factors and alleviate cytokine storm. Han et al. (2020) claimed that ophiopogonin D′, ophiopogonin D, ginsenoside Rg2, ophiopogon A, methyl ophiopogonanone A, ophiogenin-3-O-α-L-rhamnopyranosyl, ginsenoside Rb2 (1→2)-β-D-glucopyranoside, ginsenoside R0, sanchinoside Rd, ophiopogonanone E, and ginsenoside Re in Shenmai Injection showed a higher binding affinity with 3CL hydrolase. These compounds were the main effective components in the treatment of COVID-19 combined with coronary heart disease. Shenmai Injection could achieve simultaneous intervention of COVID-19 and coronary heart disease by inhibiting cytokine storms, maintaining cardiac function homeostasis, and regulating immunity and antiviral activity. It exerted a mutual influence and complex interference on a network regulatory mechanism. Fang et al. (2012) reported that Shenmai Injection could cut down the expression of TNF-α and ICAM-1 to play a protective role against lung injury. Li et al. (2020d) reported that kaempferol, ginsenoside Rh2, beta-sitosterol, stigmasterol, and deoxyandrographolide might be the main active ingredients in Shenfu Injection which cause inhibition of the SARS-CoV-2 3CL hydrolase activity and regulate ACE2. As a result, the antiviral effects, immunoregulation, and targeting of the cytokine storm by Shenfu decoction may play an important role in the treatment of critically ill patients with COVID-19 through regulating multiple signaling pathways including the AGE-RAGE signaling pathway in diabetic complications, IL17, C-type lectin receptor, and HIF-1 signaling pathways. Shenfu Injection also can reduce the seriousness of lung injury (Wang et al., 2020b). Wang et al. (2020d) found that the active compounds in Shengmai Injection, such as schisanlactone E, N-trans-feruloyltyramine, and stigmasterol, could act on CASP3, PTGS2, NOS2, NOS3, and other targets to regulate multiple signaling pathways to induce anti-inflammatory effect, immune regulation, and antishock and increase blood oxygen saturation in the treatment of COVID-19. Shengmai Injection could reduce the levels of iNOS and NF-kB, improve the peroxidation damage, and has a protective effect on acute lung in rats (Liu et al., 2009).

Kangbingdu Granules had a significant role in combating the SARS virus in 2003 and the influenza A (H1N1) virus in 2009, and thus, the formulation was recommended for pediatric prophylaxis and for adults with fever or cough and sore throat during medical diagnosis and treatment of COVID-19 in Guangdong Province. Yao et al. (2020) reported that the core active compounds such as bicuculline, luteolin, and quercetin in Kangbingdu Granules showed good affinity to SARS-CoV-2 3CL protease. They could interact with ACE2 *via* its targets PTGS2, HSP90AB1, and PTGS1 to regulate multiple signaling pathways, thereby exerting therapeutic effects on COVID-19.

Shuanghuanglian Oral Liquid is recommended by the Diagnosis and Treatment Protocol for COVID-19 in Beijing. Recent studies have revealed that Shuanghuanglian Oral Liquid has broad-spectrum antiviral and antibacterial activities, which can improve the immune response of the human body. It is an effective broad-spectrum antiviral agent. Zheng et al. (2020b) reported that the mechanism of Shuanghuanglian Oral Liquid against COVID-19 involved its active compounds including quercetin, stigmasterol, beta-sitosterol, kaempferol, neobaicalein, dihydrooroxylin A, and luteolin. These compounds might regulate related biological processes and signaling pathways by acting on key proteins involved in protein network interaction such as CASP3, TP53, MAPK8, IL6, and CCL2. The research showed that Shuanghuanglian Oral Liquid has a protective effect against acute liver failure in mice, which is mainly reflected in improving liver function, inhibiting cytokine synthesis, and reducing inflammatory injury (Wang et al., 2020f). Shuanghuanglian Oral Liquid also can inhibit the LPS-induced increase of body temperature in rats (Liang and Zhou, 2011).

The Siji Kangbingdu Mixture is a CPM recommended by the Diagnosis and Treatment Protocol for COVID-19 (trial version 2) in Shanxi Province and is applicable to mild and severe cases. Liu et al. (2020b) indicated that Siji Kangbingdu Mixture exhibited the potential to be an outstanding agent with better effectiveness and safety compared to chemical antiviral agents and predicted that the Siji Kangbingdu Mixture treatment presented a “win-win” mechanism because it was beneficial not only to symptom alleviation but also for disease resolution. The mechanism might be related to the IL17 signaling pathway and the regulation of the arachidonic acid metabolism pathway which is active in the systemic immune response and inflammation and may also interfere with SARS-CoV-2 replication by inhibiting 3CL protease.

Qingxuan Zhike Granule is recommended for COVID-19 patients with mild and moderate disease in Hunan Province, where special TCM treatment protocols for children have been introduced. Experimental studies (Ruan et al., 2010; Qiao, 2020) have shown that Qingxuan Zhike Granule could repair and improve the pathological damage caused by acute bronchitis in model rats and enhanced the bactericidal effect in rat cells. Zong et al. (2020) reported that the core compounds in Qingxuan Zhike Granules including quercetin, kaempferol, and luteolin had a similar affinity with SARS-CoV-2 3CL protease as the recommended drugs. The active ingredients in Qingxuan Zhike Granules might regulate multiple signaling pathways by acting on targets such as PTGS2, HSP90AA1, and ESR1, thereby ameliorating disease progression.

Jinzhen Oral Liquid is mainly used to treat children with bronchitis, bronchopneumonia, mycoplasma pneumonia, and viral pneumonia as well as patients with phlegm-heat cough. At present, Jinzhen Oral Liquid has become an important therapeutic aid. In addition, the academic Zhong Nanshan pointed out that Jinzhen Oral Liquid presented good antiviral and anti-inflammatory activities *in vitro* at the 24th press conference on COVID-19 prevention and control held by the Information Office of Guangdong Provincial Government. The results of modern pharmacology have shown that Jinzhen Oral Liquid presents good inhibitory or antiviral effects on the SARS virus, influenza A virus, respiratory syncytial virus, and *Mycoplasma pneumoniae* (Xiao et al., 2008; Xiao et al., 2009). Tao et al. (2020) reported that isoglabrolide, glabrolide, ebeiedinone, desoxo-glabrolid-acetate, peimisine, verticinone, imperialine, ussuriedinone, and euchrenone A5 were the active compounds of Jinzhen Oral Liquid in the treatment of COVID-19. Jinzhen Oral Liquid might inhibit the occurrence and development of the cytokine storm in COVID-19 by regulating the expression of the bromine domain containing protein 2 (Brd2), aminopeptidase N (APN), and ACE2, by interfering with the PI3K-Akt, Jak-STAT, TNF, and MAPK signaling pathways, and by inhibiting viral replication by binding with 3CL protease—thus exerting a preventive or therapeutic effect on COVID-19. *In vivo* experiments showed that Jinzhen Oral Liquid can alleviate acute lung injury by inhibiting multiple targets and blocking the NF-κB and MAPK signaling pathways (Zong et al., 2018), and it has a good antipyretic effect and is similar to the therapeutic mechanism of Xiyanping Injection (Hong et al., 2021).

Qingkailing Injection is widely used in the treatment of acute pneumonia, upper respiratory tract infection, high fever, and other diseases. Qingkailing Injection has been listed as a recommended drug by the latest version of the Diagnosis and Treatment Protocol for COVID-19 and Prevention Protocol formulated by the Tongji Hospital in Wuhan, Hubei Province. Zhang et al. (2020b) reported that the effects of Qingkailing Injection showed activity against multiple targets and multiple pathways. The active components including acacetin, syrigin, luteolin, kaempferol, quercetin, and apigenin could regulate the apoptosis pathway and the TNF pathway by acting on CASP3, CASP8, FASLG, and other targets, to achieve potential therapeutic effects on COVID-19. Gao et al. (2013) suggested that Qingkailing Injection has an antipyretic effect by decreasing the expression of 5-HT and the concentration of 4-aminobutyric acid and it can improve the metabolism of amino acids and the urea cycle. Sun et al. (2005) used LPS to cause visceral injury, and the results reflected that Qingkailing Injection had the function of protecting visceral injury as reflected through enhancing the free-radical activity and the inhibition to overoxidation by the results of detecting MDA and SOD levels.

Recent studies have suggested that the aggravation and even deaths caused by viral diseases such as COVID-19 were not only related to pulmonary virus infection, but were also closely associated with the immune dysfunction of the body (Xu et al., 2020d). The studies have shown that Bufei Huoxue Capsule could effectively improve pulmonary fibrosis, reduce the expression of inflammatory factors such as TNF-α and IL6, promote lung tissue repair, and effectively improve the immune response, which also contributes to the recovery of COVID-19 (Ma et al., 2016; Jing et al., 2017). Currently, Bufei Huoxue Capsule has been included in the recommended drug list in the Diagnosis and Treatment Protocol for COVID-19 in Guizhou Province and is mainly used for COVID-19 recovery treatment. Guo et al. (2020a) reported that the beneficial effects of Bufei Huoxue Capsule were not only associated with its antiviral and anti-inflammatory activities, but were also closely related to the regulation of immune function in the treatment of convalescent COVID-19 patients. Its key compounds including quercetin, kaempferol, 7-O-methylisomucronulatol, baicalein, and formononetin target IL6, MAPK8, PTGS2, PTGS1, and NCOA2 to regulate multiple signal pathways of TCM and play a therapeutic role in the recovery period of COVID-19.

### Common Pharmacological Actions and Mechanisms

**FIGURE 3:**
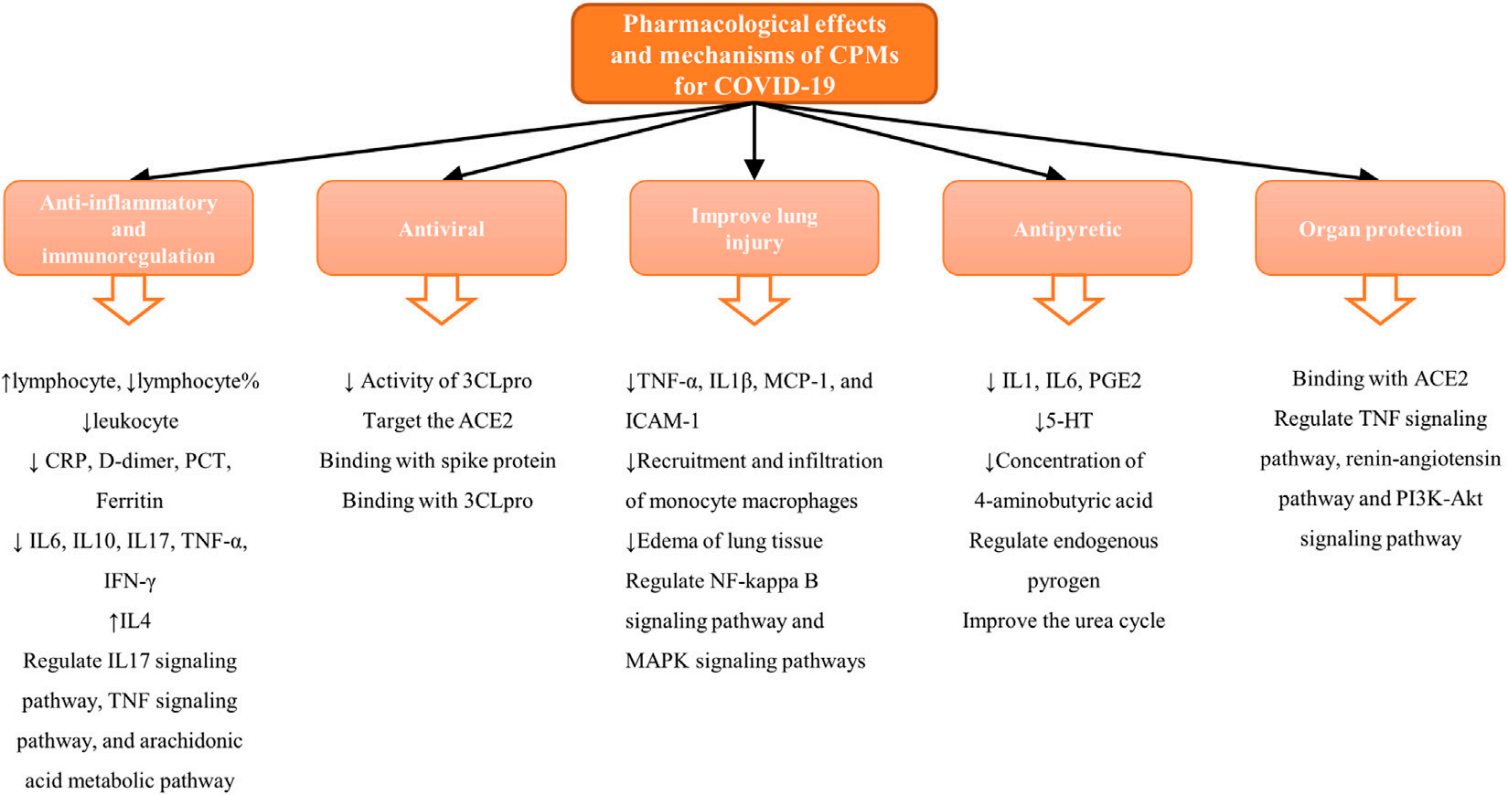
The pharmacological effects and mechanisms of CPMs for COVID-19.

**FIGURE 4:**
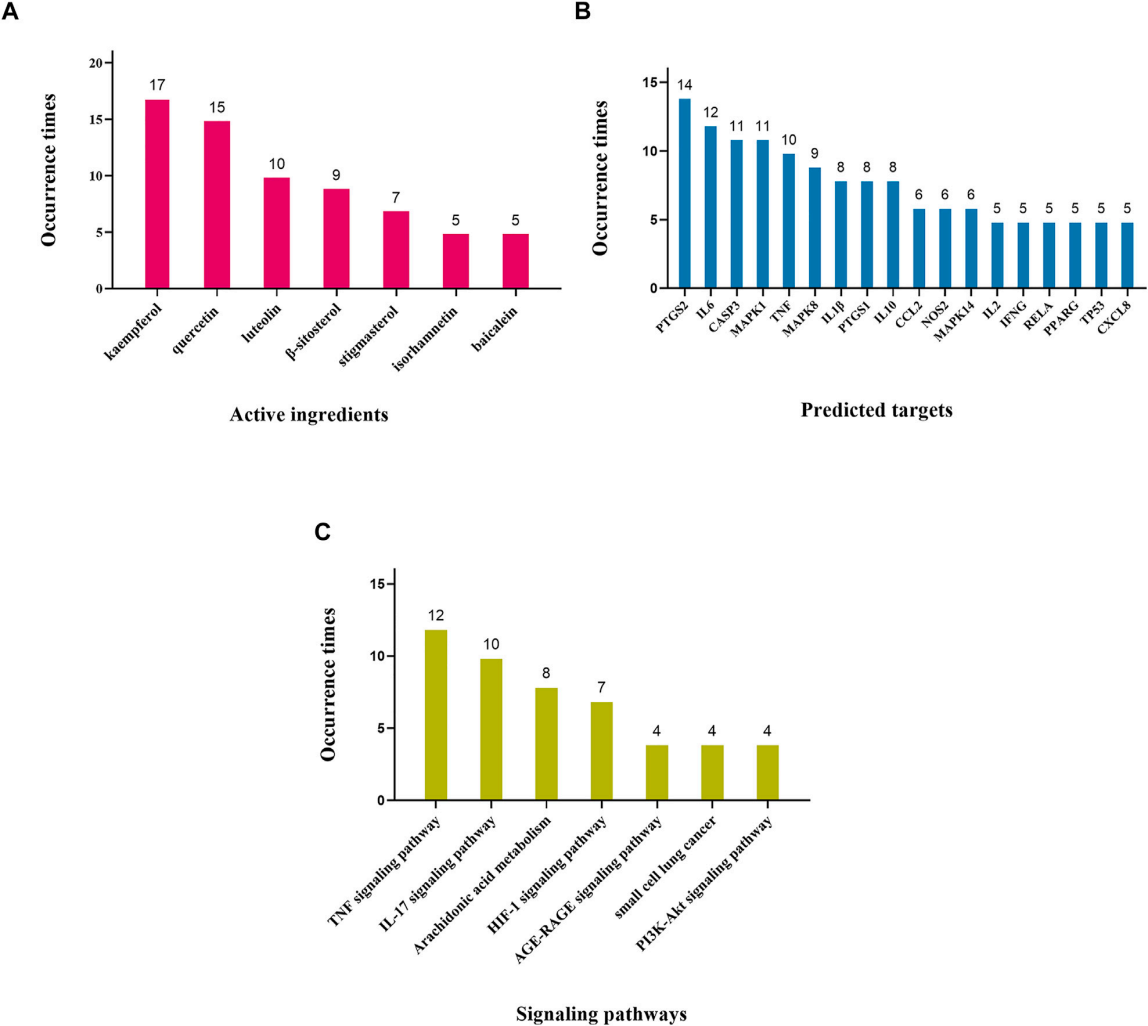
Occurrence times of potential mechanism. Note: **(A)** Occurrence times of active ingredients (frequency ≥ 5). **(B)** Occurrence times of predicted targets (frequency ≥ 5). **(C)** Occurrence times of signaling pathways (frequency ≥ 4).

On the basis of reviews and comments on the previous text, we concluded that these 22 recommended CPMs in the treatment of COVID-19 mainly act on anti-inflammatory and immunoreguation and antiviral effects and improve lung injury, antipyretic effect, and organ protection. The results are provided in Table 4 and Figure 3. In addition, we calculated the occurrence times of active ingredients, predicted targets, and signaling pathways predicted by network pharmacology and molecular docking. The results are shown in Figure 4.

**TABLE 4:** The pharmacological action of CPMs for the treatment of COVID-19.

Category	Name	Anti-inflammatory and immunoregulation	Antiviral	Improving lung injury	Antipyretic	Organ protection	Reference
Clinical efficacy has been reported	Lianhua Qingwen Capsule	++	++	++	–	–	(Li et al., 2020b); (Li et al., 2019)
	Jinhua Qinggan Granules	++	++	–	–	–	(Shen et al., 2020); (Jimilihan et al., 2020)
	Shufeng Jiedu Capsule	++	++	–	++	–	(Cao et al., 2020);
							(Xu et al., 2020c);
							(Liu et al., 2016);
	Huoxiang Zhengqi Oral Liquid	++	++	–	–	–	(Deng et al., 2020); (Du et al., 2020)
	Qingfei Paidu decoction	++	++	–	–	–	(Zhao et al., 2021)
	Xuebijing Injection	++	++	–	–	++	(Zheng et al., 2020a); (Chen et al., 2018); (Huang et al., 2020a)
	Reyanning Oral Liquid	++	–	++	–	–	(Bao et al., 2020);
							(Wang et al., 2020e)
	Jinye Baidu Granules	++	–	–	–	++	(Shi et al., 2020a)
	Reduning Injection	++	–	–	++	–	(Chen et al., 2020e); (Wang et al., 2013)
	Tanreqing Injection	++	++	++	–	–	(Kong et al., 2020b)
Potential therapeutic effects	Xiyanping Injection	++	–	++	++	++	(Cai et al., 2020);
							(Yang and Luo, 2017); (Xu et al., 2019)
	Xingnaojing Injection	++	–	–	–	–	(Xie et al., 2020)
	Shenmai Injection	++	–	++	–	++	(Han et al., 2020);
							(Fang et al., 2012)
	Shengmai Injection	++	–	++	–	–	(Wang et al., 2020d); (Liu et al., 2009)
	Shenfu Injection	++	++	++	–	–	(Li et al., 2020d); (Wang et al., 2020b)
	Kangbingdu Granules	++	++	–	–	–	(Yao et al., 2020)
	Shuang Huanglian Oral Liquid	++	–	–	++	++	(Zheng et al., 2020b); (Wang et al., 2020f);
							(Liang and Zhou, 2011)
	Siji Kangbingdu Mixture	++	++	–	–	–	(Liu et al., 2020b)
	Qingxuan Zhike Granules	++	++	–	–	–	(Qiao, 2020);
							(Zong et al., 2020)
	Jinzhen Oral Liquid	++	++	++	++	–	(Tao et al., 2020); (Hong et al., 2021); (Xiao et al., 2009)
	Qingkailing Injection	++	–	–	++	++	(Zhang et al., 2020c); (Gao et al., 2013); (Sun et al., 2005)
	Bufei Huoxue Capsule	++	++	–	++	–	(Jing et al., 2017); (Guo et al., 2020a)
++: has corresponding pharmacological actions							

Table 4 shows 22 CPMs which all have anti-inflammatory and immunomodulatory effects. Recent studies have suggested that the pathogenic mechanism of SARS-CoV-2 is closed related to inflammatory stimulation and immune dysfunction (Xu et al., 2020e). The immune system responds rapidly after SARS-CoV-2 has infected the body. CD4^+^ T lymphocytes differentiate rapidly into helper T cells Th1 and Th2, and then, Th1 secretes granulocyte–macrophage colony-stimulating factor (GM-CSF). These cytokines will induce excessive activation of CD14^+^ and CD16^+^ monocytes (Zhou et al., 2020b). When the Th1 and Th2 cells and the CD14^+^ and CD16^+^ monocytes enter the pulmonary circulation, a significant inflammatory cell infiltration, with the release of proinflammatory cytokines, chemokines, IL1β, IL6, IFN-γ, TNF-α, CCL2, CCL3, and CXCL10, will occur in the lungs, forming a “cytokine storm” causing severe pneumonia and in turn lead to lung injury, acute respiratory distress syndrome (ARDS), and multiple organ failure and even death (Huang et al., 2020a; Zhou Y. X. et al., 2020). The regulatory function of TCM on the immune system is closely related to the production and activity of T cells, B cells, NK cells, macrophages, and cytokines. From the results of COVID-19 clinical research, we can find CPMs can regulate the proportion of lymphocyte and leukocyte, significantly reduce the level of inflammation factors such as CRP, D-dimer, PCT, IL6, IL10, IL17, TNF-α, and IFN-γ, and increase the level of IL4 (Chen et al., 2020c; Qin et al., 2020). Network pharmacology research shows that the mechanism may be related to the IL17 signaling pathway, TNF signaling pathway, and arachidonic acid metabolism pathway. The biological activity of TNF is to promote inflammation, cell survival, and tissue regeneration. TNF can effectively stimulate a large amount of IgG, promote the proliferation of B cells, and activate the adhesion receptors on the surface of endothelial cells and neutrophils (Shen et al., 2020). IL17 is a proinflammatory cytokine mainly secreted by Th17 and other T cells, which exerts a major role in adaptive immunity. The IL17 signaling pathway can reduce the aggregation of macrophages and granulocytes by regulating CXCL2, CCL2, CXCL8, and other chemokines, thus reducing the inflammatory response (Noack and Miossec, 2017; Liu Y. R. et al., 2020). Ren et al. (2020c) concluded that arachidonic acid (AA) metabolism pathway is principally used to synthesize inflammatory cytokines, such as interleukin (IL), interferon (IFN), C-X-C motif chemokine (CXCL), and so on, which directly leads to the cytokine cascade reaction. Therefore, the inhibition of the AA metabolism pathway is beneficial for alleviating the “cytokine storm.”

The action mechanism of antiviral drugs is mainly through blocking a certain stage of virus replication and reproduction, such as preventing the virus from entering the host cell (Pawlos et al., 2021), inhibiting the synthesis of the virus (Kumar et al., 2021), and inhibiting the release of the virus (Martin-Sancho et al., 2021), so as to achieve the purpose of inhibiting virus reproduction and controlling virus infection. The antiviral effect of CPMs is through inhibiting the function enzyme of SARS-CoV-2 or binding with the host cell receptor. In the second part of this article, we mentioned that the main targets of anti-SARS-CoV-2 include ACE2 and 3CLpro. ACE2 plays an important role in the process of binding with the virus, while 3CLpro is related to viral transcription and replication. CPMs’ main ingredients kaempferol, quercetin, luteolin, and baicalein have better combinations with Mpro, while isorhamnetin, stigmasterol, and β-sitosterol can target the ACE2 (Ling et al., 2020; Kong et al., 2020b; Li Y. M. et al., 2020). Kaempferol glycosides are good candidates for 3a channel proteins of coronaviruses (Schwarz et al., 2014). Quercetin was proven to exert an inhibitory effect on PEDV 3CLpro (Li et al., 2020e) and interfere with multiple steps of pathogen virulence viral entry, viral replication, and protein assembly and these therapeutic effects can be enhanced by the CO administration of vitamin C. quercetin (Colunga et al., 2020). Luteolin has been reported in experimental studies to inhibit viral replication by binding to the spike protein on the surface of the coronavirus and can inhibit 3CLpro, which is required for viral infection (Ling et al., 2004). Liu et al. (2021) found that baicalein could inhibit SARS-CoV-2 3CLpro activity *in vitro* with IC50 values of 8.52 µg/ml and 0.39 µM. The effect of isorhamnetin binding to ACE2 was demonstrated by the surface plasmon resonance assay, and it was shown by *in vitro* virus experiments that isorhamnetin could inhibit SARS-CoV-2 spike pseudotyped virus entering ACE2h cells (Zhan et al., 2021). Anti-inflammatory and antiviral effects of stigmasterol and β-sitosterol were demonstrated in *in vitro* experimental studies (Petrera et al., 2014; Liao et al., 2018). In addition, kaempferol, quercetin, luteolin, baicalein, and isorhamnetin all belong to flavonoids. Studies have shown that flavonoids could interact with the S1 and S2 sites of MERS-CoV 3CLpro to play a role in antiviral effects (Jo et al., 2019; Ren et al., 2020b).

The SARS-CoV-2 infects humans mainly through the ACE2 receptor and the main invasion site is AT2 cells, resulting in severe lung injury (Kuba et al., 2005). Histopathological changes were also most common in the lungs and blood vessels. The mechanism of lung injury is mainly constituted by two aspects, the production of a variety of inflammatory cytokines by alveolar macrophages and lung epithelial cell activation, such as IL1β, IL2, IL6, IL7, IL8, IL10, tumor necrosis factor-α (TNF-α), granulocyte–macrophage colony-stimulating factor (GM-CSF), and interferon-gamma-induced protein 10 (IP-10) (Zarrilli G et al., 2021). On the other hand, upregulation of endothelial adhesion factor (VCAM, ICAM, VWF, ANG-2, and VEGF) also contributes to lung injury (Polidoro et al., 2020). Treatment of CPMs for COVID-19 is to alleviate excessive inflammatory response in lung tissue mainly by inhibiting the synthesis and release of inflammatory factors. According to the clinical and statistical data, CPMs could cut down the expression of TNF-α, IL1β, MCP-1, and ICAM-1, reduce the recruitment and infiltration of monocyte macrophages, and further reduce the edema of lung tissue to improve lung injury (Fang et al., 2012; Li et al., 2019). In addition, Jinzhen Oral Liquid can block the NF-κB and MAPK signaling pathways to improve lung injury (Zong et al., 2018).

Fever is considered to be one of the main clinical features and judgments of patients with COVID-19. After fever, immune cells activate and release pyrogenic cytokines IL1, IL6, and TNF-α, which then act on essential mediators COX-2 and PGE2 to cause hyperthermia (Ma et al., 2021). The antipyretic mechanism of CPMs is mainly achieved by reducing the cAMP content in hypothalamus and cerebrospinal fluid, inhibiting the synthesis of PGE2, and reducing the content of central neurotransmitter (Xu et al., 2019). Shufeng Jiedu Capsule and Reduning Injection exert an antipyretic effect through regulating the expression of IL1, IL6, and PGE2 and regulate endogenous pyrogen. The mechanism of antipyretic of Qingkailing Injection is to decrease the expression of 5-HT and the concentration of 4-aminobutyric acid and it can improve the metabolism of amino acids and the urea cycle (Gao et al., 2013). Baicalein, an active ingredient with high frequency in TCM, has a good antipyretic effect. Its mechanism of action is to inhibit arachidonic acid metabolism, COX and LOX, and the secretion and release of nuclear factor and cytokine (Xin, 2013).

Although the novel coronavirus pneumonia is a kind of infectious disease directed at the lungs, studies have shown that it can also damage other organs, such as the heart, nerves, brain, vessels, kidneys, and skin (Hossain et al., 2020). SARS-CoV-2 combined with ACE2 stimulated macrophages and monocytes to release proinflammatory cytokines, including IL6, NF-kB, and TNF-α, leading to inflammation-derived injurious cascades and then to multiple organ failure. Among them, ACE2 plays a key role in inflammation, which may aggravate COVID-19 through the following ways: renin–angiotensin system, including promoting the pathological changes in lung injury and involving inflammatory response (Iwasaki et al., 2021). In addition, its severity is related to the viral load and the regulation of ACE2 receptor (Loganathan et al., 2021). CPMs show high binding affinity with ACE2 playing a role in organ protection, and many active ingredients can repair organ damage. Kaempferol has a protective effect on oxidative stress-induced nerve injury and hepatocyte apoptosis by inhibiting the expression of apoptotic protein (Schwarz et al., 2014). *In vitro* test showed that baicalein had cardiovascular protective effects such as vasodilation, hypotension, myocardial protection, endothelial cell protection, and antiatherosclerosis (Xin, 2013). The TNF signaling pathway plays a role in the induction of tissue repairs, such as neuronal remyelination, cardiac remodeling, or cartilage regeneration (Noack and Miossec, 2017). The PI3K-Akt signaling pathway has a protective effect on oxidative stress and inflammatory response induced by various pharmacological media, especially playing a key role in the survival, proliferation, and apoptosis of cardiac myocytes (Li et al., 2021).

## Conclusion and Future Prospects

Among the many plagues experienced in Chinese history, TCM has played an important role in both early prevention and treatment to control the progression of the disease and improve patient prognosis (Yang, 2020b; Ren J.-l. et al., 2020). Currently, the focus and difficulties in the treatment of COVID-19 concern mainly the more severe and critical cases, which are also the main factors directly affecting mortality. The major purpose of the participation of TCM in the therapy of COVID-19 is to make full use of its advantages in the treatment of mild and moderate COVID-19 patients. For severe or critical COVID-19 patients, TCM can reduce the disease condition and decrease mortality (Yang, 2020b). However, vulnerable patient groups such as the elderly, pregnant and lactating women, infants, and patients with underlying diseases need to be especially cautious when using CPMs. Improper usage of CPMs, such as abuse or misuse, repeated drug use, overuse, drug antagonism, drug incompatibility, drug–drug interactions, and improper syndrome classification, will increase drug-induced risks (Zhang et al., 2020c); for example, when there is a repeated incompatibility in early treatment (such as taking Lianhua Qingwen Capsule and Shuanghuanglian oral liquid simultaneously), this incompatibility will influence the effects of treatment. In addition, for drugs containing ephedra (such as Lianhua Qingwen Capsule and Jinghua Qinggan Granules), doctors should pay attention to the patient’s blood pressure and heart rate.

*In vivo* experiments showed (Zhang et al., 2021) that Qingfei Paidu decoction can affect the pharmacokinetics of CYP3A substrate drugs through the inactivation of CYP3A, which leads to the risk of disease treatment with CYP3A substrate drugs such as lopinavir. Moreover, severe COVID-19 patients are prone to experience septic shock, liver and kidney dysfunction, and related underlying diseases which will reduce drug metabolism and clearance rates. For these patients, treatment regimens should avoid the use of drugs with the potential to induce liver and kidney toxicity to avoid the possibility of drug accumulation (Yang, 2020b). At the same time, it should also be noted that respiratory viral diseases, including COVID-19, may present symptoms of critical illness. Although there are corresponding treatment methods among TCM, these still present certain limitations. For these reasons, further exploration of TCM treatment of these diseases and syndromes is needed and we should actively cooperate with Western rescue strategies to improve cure rates and reduce death rates.

All of these 22 TCMs we reviewed are currently widely used clinically, and 10 have been reported in the literature to significantly improve clinical symptoms in patients with COVID-19, and a part of them have also been studied. However, due to the low number of COVID-19 cases in China and the lack of a large number of research subjects, it makes clinical research difficult. We provide a comprehensive analysis of the therapeutic effects and mechanisms of the above Chinese patent medicines, while exploring the mechanism of drug treatment, to provide some basis and clues to the clinical therapeutic effects. But this review has some limitations. At present, understanding of the mechanisms involved in TCM for the treatment of COVID-19 has been mainly achieved through virtual simulations and analyses of potential pharmacological networks and molecular docking studies. The limitations of the network pharmacology and molecular docking screening results are also in function of the complexity of TCM and of its compound composition as well as the complexity and variability of the virus. Thus, studies of composition, predicted targets, and pathways may have some limitations. Further *in vivo* and *in vitro* experiments should be carried out to verify the validation of these mechanisms to provide a scientific basis for the TCM treatment of COVID-19 and to provide an experimental basis for antiviral natural medicine research and development.

## Abbreviations

COVID-19, coronavirus disease 2019; SARS-CoV-2, severe acute respiratory syndrome coronavirus 2; TCM, traditional Chinese medicine; CPMs, Chinese patent medicines; WHO, World Health Organization; ICTV, International Committee on Taxonomy of Viruses; SARS, severe acute respiratory syndrome; MERS, Middle East respiratory syndrome; PHEIC, Public Health Emergency of International Concern; 3CLpro, 3-chymotrypsin-like protease; ACE2, angiotensin-converting enzyme 2; NSP, nonstructural proteins; ARDS, acute respiratory distress syndrome; AT2, alveolar type 2; GM-CSF, granulocyte–macrophage colony-stimulating factor; COX, cyclooxygenase; AA, arachidonic acid; IL, interleukin; IFN, interferon; C-X-C, C-X-C motif chemokine; Brd2, bromine domain containing protein 2; APN, aminopeptidase N.
