# Corrigendum: Potential Mechanisms of Action of Chinese Patent Medicines for COVID-19: A Review

**DOI:** 10.3389/fphar.2021.770125

**Published:** 2021-10-25

**Authors:** Zhi-Hua Yang, Bin Wang, Qian Ma, Lin Wang, Ya-Xin Lin, Hai-Feng Yan, Zi-Xuan Fan, Hao-Jia Chen, Zhao Ge, Feng Zhu, Hui-Jie Wang, Bao-Nan Zhang, Hai-Dong Sun, Li-Min Feng

**Affiliations:** ^1^ First Teaching Hospital of Tianjin University of Traditional Chinese Medicine, Tianjin, China; ^2^ Tianjin University of Traditional Chinese Medicine, Tianjin, China; ^3^ Department of Traditional Chinese Medicine, Hebei North University, Zhangjiakou, China; ^4^ Department of Cardiology, Traditional Chinese Medicine Hospital of Tianjin Beichen District, Tianjin, China; ^5^ Tianjin Fourth Central Hospital, Tianjin, China; ^6^ Shenzhen Hospital Futian of Guangzhou University of Chinese Medicine, Shenzhen, China; ^7^ Second Affiliated Hospital of Tianjin University of Traditional Chinese Medicine, Tianjin, China

**Keywords:** COVID-19, SARS-CoV-2, traditional Chinese medicines, Chinese patent medicines, review

Incorrect Affiliation

In the published article, there was an error in affiliation “6”. Instead of “Shenzhen Hospital Futian of Guangzhou University of Chinese Medicine, Tianjin, China”, it should be “Shenzhen Hospital Futian of Guangzhou University of Chinese Medicine, Shenzhen, China”.

We apologize for this error and state that this does not change the scientific conclusions of the article in any way. The original article has been updated.

